# Epigenetic clock analysis of diet, exercise, education, and lifestyle factors

**DOI:** 10.18632/aging.101168

**Published:** 2017-02-14

**Authors:** Austin Quach, Morgan E. Levine, Toshiko Tanaka, Ake T. Lu, Brian H. Chen, Luigi Ferrucci, Beate Ritz, Stefania Bandinelli, Marian L. Neuhouser, Jeannette M. Beasley, Linda Snetselaar, Robert B. Wallace, Philip S. Tsao, Devin Absher, Themistocles L. Assimes, James D. Stewart, Yun Li, Lifang Hou, Andrea A. Baccarelli, Eric A. Whitsel, Steve Horvath

**Affiliations:** ^1^ Department of Human Genetics, David Geffen School of Medicine, University of California Los Angeles, Los Angeles, CA 90095, USA; ^2^ Longitudinal Studies Section, Translational Gerontology Branch, National Institute on Aging, National Institutes of Health, USA. Baltimore, MD 21224, USA; ^3^ Department of Neurology, UCLA School of Medicine, University of California Los Angeles, Los Angeles, CA 90095, USA; ^4^ Department of Epidemiology, UCLA Fielding School of Public Health, University of California Los Angeles, Los Angeles, CA 90095, USA; ^5^ Geriatric Unit, Azienda Sanitaria Firenze (ASF), Florence, Italy; ^6^ Cancer Prevention Program, Division of Public Health Sciences, Fred Hutchinson Cancer Research Center, Seattle, WA 98109, USA; ^7^ Department of Medicine, New York University, New York, NY 10016, USA; ^8^ Department of Epidemiology, University of Iowa, 145 N. Riverside Drive, Iowa City, IA 52242, USA; ^9^ Department of Medicine, Stanford University School of Medicine, Stanford, CA 94305, USA; ^10^ VA Palo Alto Health Care System, Palo Alto CA 94304, USA; ^11^ HudsonAlpha Institute for Biotechnology, Huntsville, AL 35806, USA; ^12^ Department of Epidemiology, Gillings School of Global Public Health, University of North Carolina, Chapel Hill, NC 27599, USA; ^13^ Department of Genetics, School of Medicine, University of North Carolina, Chapel Hill, NC 27599, USA; ^14^ Department of Biostatistics, Gillings School of Global Public Health, University of North Carolina, Chapel Hill, NC 27599, USA; ^15^ Department of Preventive Medicine, Feinberg School of Medicine, Northwestern University Chicago, IL 60611, USA; ^16^ Robert H. Lurie Comprehensive Cancer Center, Feinberg School of Medicine, Northwestern University Chicago, IL 60611, USA; ^17^ Laboratory of Environmental Epigenetics, Departments of Environmental Health Sciences Epidemiology, Columbia University Mailman School of Public Health, New York, NY 10032, USA; ^18^ Department of Medicine, School of Medicine, University of North Carolina, Chapel Hill, NC 27599, USA; ^19^ Dept. of Biostatistics, Fielding School of Public Health, University of California Los Angeles, Los Angeles, CA 90095, USA

**Keywords:** diet, lifestyle, fish intake, alcohol intake, aging, epigenetic clock, DNA methylation

## Abstract

Behavioral and lifestyle factors have been shown to relate to a number of health-related outcomes, yet there is a need for studies that examine their relationship to molecular aging rates. Toward this end, we use recent epigenetic biomarkers of age that have previously been shown to predict all-cause mortality, chronic conditions and age-related functional decline. We analyze cross-sectional data from 4,173 postmenopausal female participants from the Women's Health Initiative, as well as 402 male and female participants from the Italian cohort study, Invecchiare nel Chianti.

Extrinsic epigenetic age acceleration (EEAA) exhibits significant associations with fish intake (p=0.02), moderate alcohol consumption (p=0.01), education (p=3×10^-5^), BMI (p=0.01), and blood carotenoid levels (p=1×10^-5^)—an indicator of fruit and vegetable consumption, whereas intrinsic epigenetic age acceleration (IEAA) is associated with poultry intake (p=0.03) and BMI (p=0.05). Both EEAA and IEAA were also found to relate to indicators of metabolic syndrome, which appear to mediate their associations with BMI. Metformin—the first-line medication for the treatment of type 2 diabetes—does not delay epigenetic aging in this observational study. Finally, longitudinal data suggests that an increase in BMI is associated with increase in both EEAA and IEAA.

Overall, the epigenetic age analysis of blood confirms the conventional wisdom regarding the benefits of eating a high plant diet with lean meats, moderate alcohol consumption, physical activity, and education, as well as the health risks of obesity and metabolic syndrome.

## INTRODUCTION

A number of behavioral lifestyle factors have been shown to relate to health, including diet, physical activity, moderate alcohol consumption, and educational attainment. For instance, diet is a modifiable behavior with the potential to mitigate chronic disease risk. Various dietary components have been reported to influence intermediate risk factors and the prevalence of age-related disease outcomes; thus there is a growing consensus regarding nutritional recommendations for maintaining optimal health. These dietary factors include whole grain & dietary fiber [1], fish & omega-3 fatty acids [2], and fruits & vegetables [3], all of which may be involved in reducing systemic inflammation [4]. Further, metabolic health has been established as one of the primary mechanisms through which diet affects health and disease [5]. Conditions such as, insulin resistance, hypercholesterolemia, hypertension, hypertryglyceremia, and systemic inflammation can be promoted by poor dietary habits and often coalesce, influencing a person's risk of atherosclerosis, diabetes mellitus, and stroke [6-8].

In addition to diet, other behaviors such as moderate alcohol consumption, increased physical activity, and higher educational attainment have all been linked to reductions in morbidity and mortality risk [9–16]. Yet, despite the strong evidence connecting lifestyle factors to health outcomes, it is still unclear whether these factors directly influence aging on a molecular level. In previous work, leukocyte telomere length (LTL) has been used to investigate the influence of lifestyle factors on replicative aging in blood [17–21]. A cross-sectional study of 2,284 participants from the Nurses’ Health Study reported that LTL was associated with BMI, waist circumference, and dietary intake of total fat, polyunsaturated fatty acids, and fiber [22]. LTL was also found to be longer among individuals who were more physically active [23, 24], as well has those with higher levels of education [25].

Another promising measure for investigating the dynamics between lifestyle and aging is the molecular biomarker known as the “epigenetic clock”. Chronological age has been shown to have a profound effect on DNA methylation levels [26–34]. As a result, several highly accurate epigenetic biomarkers of chronological age have been proposed [35–39]. These biomarkers use weighted averages of methylation levels at specific CpG sites to produce estimates of age (in units of years), referred to as "DNA methylation age" (DNAm age) or "epigenetic age". Recent studies support the idea that these measures are at least passive biomarkers of biological age. For instance, the epigenetic age of blood has been found to be predictive of all-cause mortality [40–43], frailty [44], lung cancer [45], and cognitive and physical functioning [46], while the blood of the offspring of Italian semi-super-centenarians (i.e. participants aged 105 or older) was shown to have a lower epigenetic age than that of age-matched controls [47]. Further, the utility of the epigenetic clock method using various tissues and organs has been demonstrated in applications surrounding Alzheimer's disease [48], centenarian status [47, 49], development [50], Down syndrome [51], frailty [44], HIV infection [52], Huntington's disease [53], obesity [54], lifetime stress [55], menopause [56], osteoarthritis [57], and Parkinson's disease [58].

However, relatively little is known about the relationship between epigenetic aging rates and lifestyle factors, such as diet, alcohol consumption, physical activity, and educational attainment. Here, we investigate these relationships by leveraging blood DNA methylation data from two large epidemiological cohorts. In our primary analysis, we use data from older women within the Women's Health Initiative (WHI) to examine the relationships between epigenetic age acceleration in blood and dietary variables, education, alcohol, and exercise. In our secondary analysis, we sought to validate the results in the Invecchiare nel Chianti (InCHIANTI) Study, which is a population-based prospective cohort study of residents ages 21 or older from two areas in the Chianti region of Tuscany, Italy.

Since our study revealed that metabolic syndrome is associated with accelerated epigenetic aging, we also carried out a post-hoc analysis that evaluated the effect of metformin, which is a widely-used medication against type 2 diabetes.

## RESULTS

### Sample characteristics

The WHI sample consisted of 4,173 postmenopausal women including 2,045 Caucasians, 1,192 African Americans, and 717 Hispanics. Chronological age ranged from 50-82 years (mean=64, s.d.=7.1). The InCHIANTI sample was composed of 402 participants from a European (Italian) population, including 178 men (44%) and 229 women (56%). We used the most current cross-sectional wave for this cohort, and at that time-point participants ranged in age from 30 to 100 years (mean=71, s.d.=16). Additional details on participant characteristics can be found in the Methods and in Table 1.

**Table 1:** Characteristics of the WHI and InCHIANTI samples

			WHI		InCHIANTI	
			Count	Percent	Count	Percent
**Ethnic**	American Indian or Alaskan Native		56	1%		
	Asian or Pacific Islander		140	3%		
	Black or African-American		1277	28%		
	Hispanic/Latino		784	17%		
	White (not of Hispanic origin)		2196	49%		
	Other		37	1%		
**WHI data set**	BA23		2098	47%		
	AS315		2392	53%		
**Sex**	Male				178	44%
	Female				229	56%
**Current smoker**	Nonsmoker		4027	90%	367	90%
	Smoker		439	10%	40	10%
**Education**	< Primary		43	1%	80	20%
	> Primary		154	3%	154	38%
	> Lower secondary		293	7%	91	22%
	> Upper secondary		2588	58%	62	15%
	> Higher		1393	31%	20	5%
**Physical activity**	Active		894	20%	329	81%
	Inactive		3572	80%	78	19%
			**Mean**	**SD**	**Mean**	**SD**
**Diet**	Total energy, kcal	kcal/day	1641	777	2069	573
	Carbohydrate	% kcal	49.0	9.1	52.4	6.9
	Protein	% kcal	16.5	3.3	15.8	2.0
	Fat	% kcal	34.6	8.1	30.9	5.5
	Red meat	serv/day	0.8	0.7	1.1	0.5
	Poultry	serv/day	0.4	0.3	0.2	0.2
	Fish	serv/day	0.3	0.3	0.2	0.2
	Dairy	serv/day	1.6	1.3	2.8	1.8
	Whole grains	serv/day	1.2	0.9		
	Nuts	serv/day	0.2	0.3	0.0	0.1
	Fruits	serv/day	1.7	1.3	1.9	0.9
	Vegetables	serv/day	1.9	1.3	1.6	0.8
	Alcohol	g/day	3.6	9.6	12.7	14.9
	**Measurements**	IEAA	years	0.0	4.7	0.2	4.6
EEAA		years	0.0	6.0	-0.2	6.5
C-reactive protein		mg/L	5.2	6.6	3.9	7.4
Insulin		mg/dL	57.1	115.3		
Glucose		mg/dL	106.3	38.0	93.0	21.3
Triglycerides		mg/dL	146.4	85.6	122.7	81.5
Total cholesterol		mg/dL	228.4	42.7	207.2	36.6
LDL cholesterol		mg/dL	144.9	39.7	125.5	32.1
HDL cholesterol		mg/dL	54.0	14.3	57.6	15.7
Creatinine		mg/dL	0.8	0.2	0.9	0.4
Systolic blood pressure		mmHg	130.0	18.0	129.3	19.8
Diastolic blood pressure		mmHg	75.8	9.4	77.2	10.3
Waist / hip ratio		cm/cm	0.8	0.1	0.9	0.1
BMI		cm/m2	29.7	6.0	27.0	4.3

The cohort samples are listed for each column and variables of interest are listed for each row. The upper portion of the table correspond to categorical variables and are described using counts and percentages; the lower portion of the table displays continuous variables which are described using means and standard deviations (SD).

### Dietary and metabolic associations with measures of age acceleration

Here we leverage two distinct measures of epigenetic age acceleration which are based on different sets of CpGs: *intrinsic* epigenetic age acceleration (IEAA), and *extrinsic* epigenetic age acceleration (EEAA) (Methods). Epigenetic age acceleration is broadly defined as the epigenetic age left unexplained by chronological age, where intrinsic and extrinsic denote additional modifications to this concept. In addition to adjusting for chronologic age, IEAA also adjusts the epigenetic clock for blood cell count estimates, arriving at a measure that is unaffected by both variation in chronologic age and blood cell composition. EEAA, on the other hand, integrates known age-related changes in blood cell counts with a blood-based measure of epigenetic age [37] before adjusting for chronologic age, making EEAA dependent on age-related changes in blood cell composition. In essence, IEAA can be interpreted as a measure of cell-intrinsic aging and EEAA as a measure of immune system aging, where for both, a positive value indicates that the epigenetic age of an individual (organ or tissue) is higher than expected based on their chronological age—or that the individual is exhibiting accelerated epigenetic aging. We find that IEAA is only moderately correlated with EEAA (r=0.37), and that measurements on the same individuals at different time points (mean difference 3.0 years between visit dates) showed moderately strong correlations (IEAA r=0.70, EEAA r=0.66).

**Figure 1:**
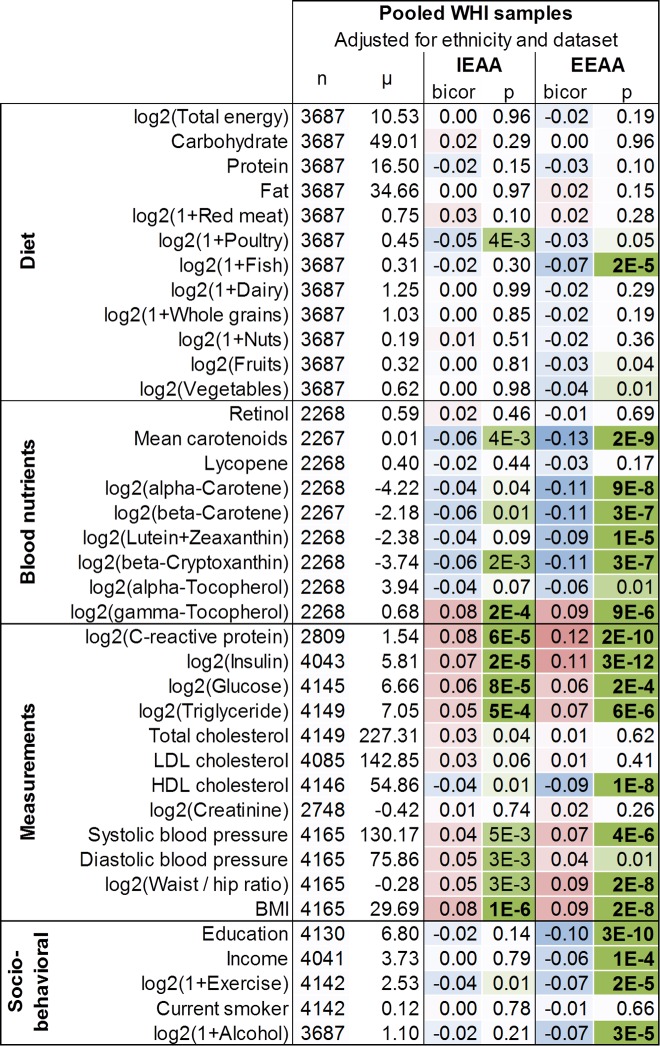
Marginal correlations with epigenetic age acceleration in the WHI Correlations (bicor, biweight midcorrelation) between select variables and the two measures of epigenetic age acceleration are colored according to their magnitude with positive correlations in red, negative correlations in blue, and statistical significance (p-values) in green. Blood biomarkers were measured from fasting plasma collected at baseline. Food groups and nutrients are inclusive, including all types and all preparation methods, e.g. folic acid includes synthetic and natural, dairy includes cheese and all types of milk, etc. Variables are adjusted for ethnicity and dataset (BA23 or AS315).

We first used a robust correlation test to relate our two measures of epigenetic aging (IEAA and EEAA) to select reported dietary exposures, blood nutrient levels, cardiometabolic plasma biomarkers, and lifestyle factors, designating a Bonferroni-corrected significance threshold of α=7×10^-4^ (Figure 1). The correlation test results for specific racial/ethnic groups are presented in Supplementary Figure 1 and select marginal associations are shown as bar plots in Supplementary Figure 2. Pairwise correlations between dietary variables, metabolic biomarkers, and lifestyle factors are presented in Supplementary Figure 3.

EEAA exhibits weak but statistically significant correlations with fish intake (r=-0.07, p=2×10^-5^), alcohol consumption (r=-0.07, p=3×10^-5^, Supplementary Figure 4), plasma levels of mean carotenoids (r=-0.13, p=2×10^-9^), alpha-carotene (r=-0.11, p=9×10^-8^), beta-carotene (r=-0.11, p=3×10^-7^), lutein+zeaxanthin (r=-0.9, p=1×10^-5^), beta-cryptoxanthin (r=-0.11, p=3×10^-7^), gamma-tocopherol (r=0.09, p=9×10^-6^), triglyceride (r=0.7, p=6×10^-6^), C-reactive protein (CRP, r=0.12, p=2×10^-10^), insulin (r=0.11, p=3×10^-12^), HDL cholesterol (r=-0.09, p=2×10^-8^), glucose (r=0.06, p=2×10^-4^), systolic blood pressure (r=0.07, p=4×10^-6^), waist-to-hip ratio (WHR, r=0.09, p=2×10^-8^), BMI (r=0.09, p=2×10^-8^), education (r=-0.10, p=3×10^-10^), income (r=-0.06, p=1×10^-4^), and exercise (r=-0.07, p=2×10^-5^, Figure 1). In contrast, the intrinsic epigenetic aging rate exhibits weaker correlations with dietary variables and lifestyle factors: IEAA is only associated with BMI (r=0.08, p=1×10^-6^), and plasma levels of gamma-tocopherol (r=0.08, p=2×10^-4^), CRP (r=0.08, p=6×10^-5^), insulin (r=0.07, p=2×10^-5^), glucose (r=0.06, p=8×10^-5^), and triglyceride levels (r=0.05, p=5×10^-4^, Figure 1).

### Meta-analysis of multivariable linear models link epigenetic age acceleration to diet

#### Associations with EEAA

**Figure 2:**
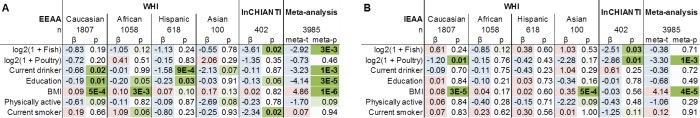
Meta-analysis of multivariable linear models of EEAA and IEAA in the WHI and InCHIANTI EEAA (panel **A**) and IEAA (panel **B**) were regressed on potential confounding factors, fish and poultry intake, and current drinker status for the ethnic strata with sufficient sample sizes (n>100). Individual columns correspond to coefficient estimates (β) colored blue or red for negative and positive values respectively, and p-values (p) colored in green according to magnitude of significance, with the exception of the last two columns which denote Stouffer's method meta-t and meta-p values. Models are adjusted for originating dataset (WHI BA23 or AS315) and for sex (InCHIANTI).

We have recently shown that ethnicity relates to epigenetic aging rates: e.g. Hispanics have lower levels of IEAA compared to other ethnic groups [59]. Given the potential for confounding by sociodemographic and lifestyle factors, we used Stouffer's method to meta-analyze multivariate linear models, stratified by racial/ethnic group, in order to re-examine the suggestive associations from our marginal correlation analysis. After adjusting for sex and dataset (Figure 2A), we find that lower EEAA is significantly associated with greater intake of fish (t_meta_=-2.92, p_meta_=0.003), higher education (t_meta_=-4.14, p_meta_=3×10^-5^), lower BMI (t_meta_=4.86, p_meta_=1×10^-6^), and current drinker status (t_meta_=-3.23, p_meta_=0.001). However, we find no association for current smoking status, and poultry intake, and only a trend toward association with physical activity (t_meta_=-1.70, p_meta_=0.09). In the subset of WHI participants with circulating carotenoid measurements, we also find that mean carotenoid levels are associated with EEAA (t_meta_=-4.34, p_meta_=1×10^-5^, Supplementary Figure 5A).

**Figure 3:**
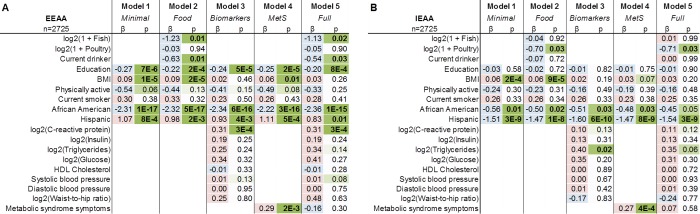
Multivariate linear models of EEAA and IEAA with and without biomarkers in the WHI EEAA (panel **A**) and IEAA (panel **B**) were regressed on potential confounding factors, fish and poultry intake and current drinker status, and select biomarkers. Individual columns list the corresponding coefficient estimates (β) and p-values (p) for each fitting. Coefficients are colored according to sign (positive = red, negative = blue) and significance according to magnitude (green). Models 1 through 5 correspond to a minimal model, a model including dietary intake variables, a model including potential explanatory biomarkers, a model including number of metabolic syndrome symptoms and a complete model with all of the variables above, respectively. Models are adjusted for originating dataset (BA23 or AS315).

Multivariate linear models were used to examine whether variations in cardiometabolic biomarkers and/or the number of symptoms for metabolic syndrome accounted for any of the associations between EEAA and lifestyle factors. The inclusion of biomarkers in an unstratified model shows that EEAA positively relates to CRP (log2, β=0.31, p=3×10^-4^, Figure 3A, model 3) and that this is accompanied by a concomitant diminishing in the effect size of BMI (67% decrease in coefficient magnitude, Figure 3A, model 2 vs. model 5), suggesting that higher CRP may partially explain the positive association between BMI and EEAA. When metabolic syndrome (MetS) was included in the model, results showed that higher EEAA is positively associated with the number of metabolic syndrome symptoms (β=0.29, p=0.002, Figure 3A, model 4). In the subset of participants with both biomarker and carotenoid measurements, EEAA was negatively associated with mean carotenoid levels (β=-1.10, p=1×10^-4^) while appearing to diminish associations with biomarkers (Supplementary Figure 6A, model 5).

Additionally, we find that for the small subset of individuals for whom we have EEAA measurements at two time points (n=239, mean time interval = 2.7 years), increase in BMI (β=0.40, p=0.002) but not initial BMI (β=-0.01, p=0.81) is significantly associated with increased EEAA (higher follow-up EEAA after adjusting for the initial EEAA, dataset, and ethnicity).

#### Associations with IEAA

We conducted an analogous meta-analysis of ethnically-stratified linear models of IEAA and found that lower IEAA was significantly associated with poultry intake (t_meta_=-3.30, p_meta_=0.001) and lower BMI (t_meta_=4.14, p_meta_=4×10^-5^), after adjusting for potential confounders (Figure 2B). In the subset of participants with measured carotenoids, IEAA was significantly associated with mean carotenoid levels (t_meta_=-2.47, p_meta_=0.01, Supplementary Figure 5B). When regressed on clinical biomarkers IEAA was significantly associated with triglycerides (log2, β=0.40, p=0.02, Figure 3B, model 3). Their inclusion diminished the association between IEAA and BMI (60% decrease in coefficient magnitude, Figure 3B, model 2 vs. model 5). Number of metabolic syndrome symptoms was also significantly associated with IEAA (β=0.27, p=4×10^-4^, Figure 3B, model 4), and diminished the association between IEAA and BMI by 50%. In the subset of WHI participants with circulating carotenoid measurements, we find a trend toward association between IEAA and mean carotenoid levels (β=-0.40, p=0.07, Supplementary Figure 6B, model 5). Finally, in the participants with epigenetic profiling at two time points, increase in BMI (β=0.22, p=0.03) but not initial BMI (β=-0.22, p=0.44) is significantly associated with increased IEAA (higher follow-up IEAA after adjusting for the initial IEAA, dataset, and ethnicity).

### Metformin and epigenetic age acceleration

Considering the significant association between our measures of epigenetic aging and markers of diabetes, we investigated the potential of metformin to modulate epigenetic aging. As shown in Supplementary Figure 7, women in the No Metformin group were the oldest, with mean ages around 65 years, compared to mean ages around 64 years for the Metformin Now group and 62.5 years for the Metformin Future group. Those in the No Metformin group also appeared to have a higher proportion of college graduates compared to the other two groups, whose members tended to have lower educational attainment. The two Metformin groups had much higher proportions of Non-Hispanic blacks, and somewhat higher proportions of Hispanics. As expected, women who were prescribed Metformin had the highest fasting insulin, fasting glucose, and HOMA-IR levels, with current Metformin users being the highest of all. Similarly, among Metformin users, fasting insulin, fasting glucose, and HOMA-IR levels were inversely associated with timing of Metformin initiation. This suggests that 1) women who had been on Metformin the longest at the time of blood draw were the most insulin resistant—arguably because they were long-time diabetics—and 2) among women who would use Metformin in the future, those who would receive prescriptions sooner—who may already have pre-diabetes or diabetes at the time of blood draw—were more insulin resistant than women who were prescribed Metformin later.

As shown in Supplementary Figure 8, when examining the full sample, IEAA and EEAA were highest for women in the Metformin Now group, although differences between Metformin Now and Metformin Future were not significant. Further, the timing of Metformin prescription relative to the timing of blood draw was not associated with either IEAA or EEAA. Overall, these results held for IEAA when restricting the sample to only Non-Hispanic whites, Non-Hispanic blacks, or women at least 65 years of age. Results for EEAA held when restricting the sample to only Hispanics, women less than 65 years of age, or those with fasting glucose levels under 126 mg/dL. Nevertheless, there were no strata in which women in the Metformin Now group had significantly lower IEAA or EEAA than women in either the No Metformin or the Metformin Future group.

Given that Metformin may not be effective for all or that not all women prescribed Metformin may comply with their physician's recommendations, we compared future Metformin users to current Metformin users who had fasting glucose levels under 140 mg/dL (Supplementary Figure 9). Similar to Supplementary Figure 8, no significant differences were found for the two metformin groups, and in most cases those currently on Metformin had somewhat higher IEAA and EEAA than those who would be prescribed Metformin in the future.

Finally, when using women with repeat blood draws to compare the change in IEAA and EEAA for those who started Metformin between the two visits to those who didn't (Supplementary Table 1), we find that, while those who start Metformin appear to have lower IEAA (β=-0.79), the difference is not significant (P=0.46). Results for EEAA show very little difference between those who start Metformin between first and second blood draws, and those who don't (β=-0.003, P=0.99). Given that we only have 11 women who start Metformin, our null results, particularly for IEAA, may be due to a lack a power. Thus, in moving forward, this analysis may be worth revisiting in a randomized control trial or after acquiring larger sample sizes.

## DISCUSSION

**Figure 4:**
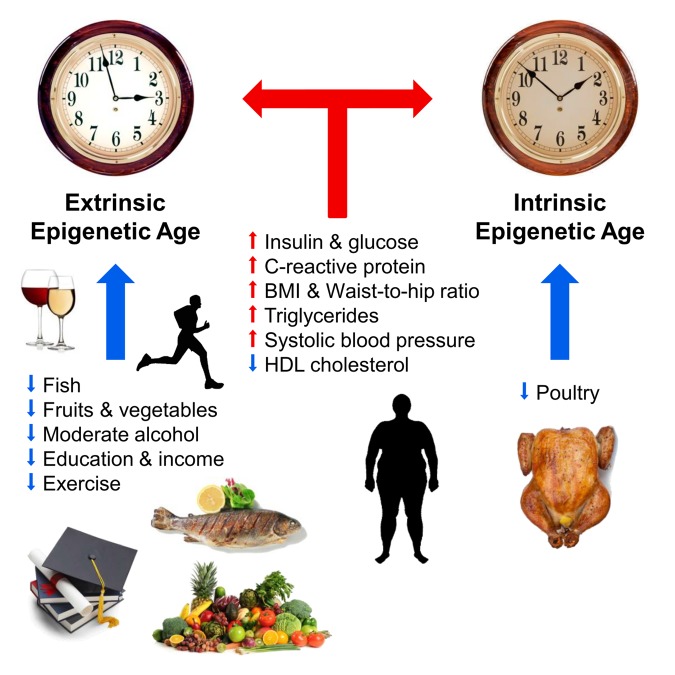
Pictorial summary of our main findings The blue and red arrows depict anti-aging and pro-aging effects in blood respectively. The two clocks symbolize the extrinsic epigenetic clock (enhanced version of the Hannum estimate) and the intrinsic epigenetic clock (Horvath 2013) which are dependent and independent of blood cell counts, respectively.

To our knowledge, this is the first study to examine associations between lifestyle factors and measures of epigenetic age acceleration in blood. Our main findings are summarized graphically in Figure 4. Overall, our dietary results are consistent with some of the current Dietary Guidelines for Americans [60, 61], reflecting potential health benefits associated with higher intake of fish, poultry, and fruits and vegetables. The weak correlations between dietary factors and epigenetic aging rates probably reflect that a relatively large proportion of the variance in aging rates (around 40 percent) is explained by genetic factors [38, 59, 62]. We find that education, physical activity, low body mass index are associated with a slow extrinsic age acceleration both in univariate correlation tests (Figure 1) and in multivariate regression models (Figure 2A, 3A). However, consistent with our previous work, smoking status was not associated with epigenetic age acceleration [45], which highlights that not every poor lifestyle choice is associated with an increased epigenetic aging effect in blood tissue.

### EEAA, inflammation, and metabolic functioning

The age-related changes in immune functioning and inflammation are believed to contribute to increased susceptibility of a wide range of diseases later in life, including diabetes, some cancers, cardiovascular, neurodegenerative, auto-immune, and infectious diseases [63, 64]. In our analysis, EEAA, a biomarker which explicitly incorporates aspects of immune system aging such as age-related changes in blood cell counts, was associated with cardiometabolic biomarkers, fish, fruit, vegetable, and alcohol intake.

Our finding that fish intake was negatively associated with EEAA is consistent with prospective studies suggesting that fish consumption is protective against various age-related diseases [65–67]. The benefits of fish intake may be mediated in part through the omega-3 fatty acids, eicosapentaenoic acid (EPA) and docosahexaenoic acid (DHA), which stimulate the synthesis of anti-inflammatory cytokines [68]. This is further supported by our finding that CRP—a well-known marker of inflammation—was the most significant explanatory biomarker of EEAA. This suggests that one reason higher fish consumption may lower EEAA is because it has beneficial anti-inflammatory or metabolic effects. The consensus between these associations also appears to converge on MetS as a potential mediating factor; this was further supported by our results showing that the number of MetS characteristics significantly relates to EEAA. Though CRP is not included in most MetS diagnostic criteria, the association between the two has been previously established [69].

We also find that alcohol consumption was negatively associated with EEAA even after adjusting for potential confounders such as socioeconomic status; this is consistent with prospective studies which have identified light to moderate alcohol intake as a protective factor against all-cause and CHD-related mortality [70, 71] and is supported by a recent publication that also found an inverse association between epigenetic age and alcohol intake in Caucasian and African American individuals (n=656, n=180, respectively) [72]. In our study, we find that the potential benefits of alcohol consumption are observed using a threshold of more than one serving per month, though the effect size of this variable was also stable when adding weekly and daily intake levels (Supplementary Figure 4A-D). The association appears to be driven by wine consumption though there is also a trend towards association with beer (Supplementary Figures 4E-H). This is consistent with other studies have suggested that wine may have added benefits compared to light alcohol consumption [73]. This finding may also be related to the anti-inflammatory effects of light alcohol consumption, which are associated with decreased circulating levels of inflammatory markers such as IL-6 and CRP [74]. Alternatively, this may be the result of reverse causation, whereby individuals suffering from health issues abstain from alcohol consumption [75], though interventional studies support a causal protective effect of moderated alcohol intake on cardiovascular blood biomarkers [76].

Though EEAA only trended toward association with reported fruit and vegetable intake, we find significant associations with blood carotenoid levels, which are quantitative surrogates of fruit and vegetable intake; this is likely a reflection of the bias and inaccuracy of self-reported diet. This is in agreement with the wide range of literature supporting the protective effects of high fruit and vegetable intake against age-related diseases CHD [77, 78], stroke [79], type-2 diabetes [80], breast cancer[81], and all-cause mortality [82]. The association between fruit and vegetables with aging of the blood immune system may be partially mediated by anti-inflammatory [83, 84] and cardiometabolic effects, however it is interesting to note that the explanatory power of mean carotenoid levels remained even after including the other explanatory factors into the model, suggesting the possibility of independent anti-aging mechanisms (Supplementary Figure 6A, model 5).

Our results for EEAA also share similarities with previously reported findings showing that LTL relates to BMI [22], metabolic factors, vegetable consumption [85], and dietary intake of foods high in omega-3 fatty acids [86]. This agreement is likely a reflection of the shared immunological basis, which is supported by the weak negative correlation between EEAA and age-adjusted LTL. In contrast, IEAA is not significantly associated with LTL, supporting the idea that these measures represent different aspects of aging.

### Intrinsic epigenetic aging and metabolic health

Results showed that BMI has a positive association with IEAA (Figure 3B, model 2). The statistically significant but weak correlations between BMI and epigenetic age acceleration in blood (r<0.10) are much smaller than those we recently reported for human liver (r=0.42) [54], suggesting that associations between aging signatures and risk factors may vary in strength depending on the tissue, and may be stronger in organs/tissues most affected by the risk. Interestingly, IEAA was also associated with number of metabolic syndrome characteristics, suggesting a role in tracking metabolic aging processes (Figure 3B, model 4).

We did find that reported poultry intake was negatively associated with IEAA, even after adjusting for potential confounders and explanatory factors (Figure 3B, model 5). Given the relative inert behavior of IEAA, the mechanism by which poultry may affect aging is unclear.

### Lack of an effect of metformin

Metformin, which is a first-line medication for the treatment of type 2 diabetes, has garnered substantial interest by aging researchers [87]. The fact that we did not detect an anti-aging effect of metformin in our study could be due to the following factors: a) it could reflect the limitations/biases of an observational study, b) it could reflect the limited sample size, or c) that metformin does not affect epigenetic aging rates of blood tissue. Future randomized controlled trials should revisit the question whether metformin affects epigenetic aging rates in blood or other tissues.

### Generalization to the InCHIANTI

Our results from the InCHIANTI show some validation of our findings from the WHI: fish intake was related to EEAA, and poultry was related to IEAA. Associations with available biomarkers of cardiometabolic health, however, were not found to be validated, and in a few cases were reversed in directions, within the InCHIANTI (data not shown).

The discrepancies between the WHI and InCHIANTI cohorts may be due to numerous differences in the study population (cultural, demographic, genetic, health status, Table 1) and data collection methodology (dietary assessment). Despite being younger, on average, US participants from the WHI had higher body mass indexes (BMI), and worse metabolic health than their Italian counterparts—as indicated by their greater prevalence of metabolic syndrome (23% in the WHI versus 7.6% in the InCHIANTI).

The InCHIANTI study is also arguably underpowered (n=402) when it comes to detecting the weak associations with epigenetic age acceleration. According to sample size calculations (*PASS* software), we find that n=1820 samples are needed to provide 80% power to detect a correlation of r=0.08 at a two-sided significance level of α=0.01. Similarly, n=1163 samples are needed to detect a correlation of r=0.10.

### Limitations

While our study of the WHI benefits from having a relatively large sample size, associations with epigenetic aging may not be detectable in smaller studies given the weak effect sizes observed here. This situation is exacerbated by self-reported lifestyle habits which are notorious for bias and inaccuracy, limiting their ability to represent true lifestyle habits and potentially producing false negative results. Further, as evidenced by our results from the ethnic strata and the InCHIANTI, studies conducted in different ethnic populations may not be entirely consistent due to fundamental differences in age, diet, culture, and demographics. There were several potential limitations to this study, which include the assumption of non-confounding from unmeasured variables such as existing patient co-morbidities and the assumption of accuracy and long-term consistency of reported dietary habits. This is the first longitudinal study to show that an increase in BMI is associated with an increase in epigenetic age acceleration but larger longitudinal studies will be needed to dissect causal relationships between epigenetic aging rates and dietary measures, education, exercise, and lifestyle factors.

### Conclusions about epigenetic age acceleration

Our large sample size (n>4500) provides sufficient statistical power for one of our main conclusions: diet has only a weak effect on epigenetic aging rates in blood. These findings will be valuable for researchers who plan to use epigenetic biomarkers in dietary intervention studies. The wide range of associations found with EEAA suggest that immune system aging may be closely linked to conventional notions of metabolic health and may be sensitive to variations in environment and lifestyle. In contrast, IEAA has few associations, which is consistent with the hypothesis that cell-intrinsic aging remains relatively stable, more likely being determined by an intrinsic aging or developmental process under genetic control. Further, using longitudinal data in the WHI, we found that change in both EEAA and IEAA are significantly associated with change in BMI, suggesting that both modes of epigenetic aging may respond to changes in lifestyle, at least with respect to change in obesity. Overall, our results are consistent with previous literature supporting the protective effects of fish, poultry and alcohol consumption, exercise, education, as well as the risk of obesity and dyslipidemia.

## METHODS

### Estimation of DNA methylation age

DNAm age (also referred to as epigenetic age) was calculated from human samples profiled with the Illumina Infinium 450K platform, described in detail in [38]. Briefly, the epigenetic clock is defined as a prediction method of age based on the DNAm levels of 353 CpGs. Predicted age, referred to as DNAm age, correlates with chronological age in sorted cell types (CD4+ T cells, monocytes, B cells, glial cells, neurons), tissues, and organs, including: whole blood, brain, breast, kidney, liver, lung, saliva [38].

We also applied the Hannum measure of DNAm age based on 71 CpGs which was developed using DNA methylation data from blood [37].

Despite high correlations, DNAm age estimates can deviate substantially from chronological age at the individual level, and adjusting for age we can arrive at measures of epigenetic age acceleration as described in the following.

### Estimation of Intrinsic and Extrinsic Epigenetic Age Acceleration (IEAA, EEAA)

In this article, we consider two measures of epigenetic age acceleration. These measures, referred to as intrinsic and extrinsic age acceleration only apply to blood. IEAA is derived from the Horvath measure of DNAm age based on 353 CpGs [38], and is defined as the residual resulting from regressing Horvath DNAm age on chronological age and estimates of plasmablasts, naive and exhausted CD8+ T cells, CD4+ T cells, natural killer cells, monocytes, and granulocytes. Thus, IEAA is independent of chronological age and most of the variation in blood cell composition. IEAA is meant to capture cell-intrinsic properties of the aging process that exhibits preservation across various cell types and organs.

EEAA can be interpreted as an enhanced version of the Hannum measure of DNAm age based on 71 CpGs [37]. EEAA up-weights the contributions of age related blood cell counts [43]. Specifically, EEAA is defined using the following three steps. First, we calculated the epigenetic age measure from Hannum et al, which already correlated with certain blood cell types [40]. Second, we increased the contribution of immune blood cell types to the age estimate by forming a weighted average of Hannum's estimate with 3 cell types that are known to change with age: naïve (CD45RA+CCR7+) cytotoxic T cells, exhausted (CD28-CD45RA-) cytotoxic T cells, and plasmablasts using the Klemera Doubal approach [88]. The weights used in the weighted average are determined by the correlation between the respective variable and chronological age. The weights were chosen on the basis of the WHI data and the same (static) weights were used for all data sets. Finally, EEAA was defined as the residual variation resulting from a univariate model regressing the resulting age estimate on chronological age. Thus, EEAA tracks both age related changes in blood cell composition and intrinsic epigenetic changes.

In a recent large scale meta-analysis involving over 13 thousand subjects from 13 cohorts, we have shown that both IEAA and EEAA are predictive of mortality, independent of chronological age, even after adjusting for additional risk factors, and within the racial/ethnic groups that we examined (Caucasians, Hispanics, African Americans) [43].

IEAA and EEAA can be obtained from the online DNAm age calculator (http://labs.genetics.ucla.edu/horvath/dnamage/), where they are denoted as *AAHOAdjCellCounts* and *BioAge4HAStaticAdjAge*, respectively.

### Dietary assessment in the Women's Health Initiative (WHI)

Participants were selected from the WHI, a national study that began in 1993 and enrolled postmenopausal women between the ages of 50-79 years into either randomized clinical trials (RCTs) or into an observational study [89] (more details available in Supplementary Information). Participants completed self-administered questionnaires at baseline which provided personal information on a wide range of topics, including sociodemographic information (age, education, race, income), and current health behaviors (recreational physical activity, tobacco and alcohol exposure, and diet). Participants also visited clinics at baseline where certified Clinical Center staff collected blood specimens and performed anthropometric measurements including weight, height, hip and waist circumferences, and systolic and diastolic blood pressures; body mass index and waist to hip ratio were calculated from these measurements (Table 1).

Dietary intake levels were assessed at baseline using the WHI Food Frequency Questionnaire [90]. Briefly, participants were asked to report on dietary habits in the past three months, including intake, frequency, and portion sizes of foods or food groups, along with questions concerning topics such as food preparation practices and types of added fats. Nutrient intake levels were then estimated from these responses. For current drinker, we use the threshold of more than one serving equivalent (14g) within the last 28 days.

### Estimation of blood cell counts based on DNA methylation levels

We estimate blood cell counts using two different software tools. First, Houseman's estimation method [91], which is based on DNA methylation signatures from purified leukocyte samples, was used to estimate the proportions of CD8+ T cells, CD4+ T, natural killer, B cells, and granulocytes (also known as polymorpho-nuclear leukocytes). Second, the advanced analysis option of the epigenetic clock software [38, 52] was used to estimate the percentage of exhausted CD8+ T cells (defined as CD28-CD45RA-) and the number (count) of naïve CD8+ T cells (defined as CD45RA+CCR7+). We and others have shown that the estimated blood cell counts have moderately high correlations with corresponding flow cytometric measures [91, 92]. For example, flow cytometric measurements from the MACS study correlate strongly with DNA methylation based estimates: r=0.63 for CD8+T cells, r=0.77 for CD4+ T cells, r=0.67 for B cell, r=0.68 for naïve CD8+ T cell, r=0.86 for naïve CD4+ T, and r=0.49 for exhausted CD8+ T cells [92].

### Blood biomarkers and DNA methylation in the WHI

Two separate subsamples were aggregated for our study within the WHI (BA23 and AS315). Both had baseline blood specimens collected after an overnight fast in EDTA tubes and stored at -70C. These samples were processed at the WHI core laboratory and select nutrient and cardiovascular biomarkers were measured including lycopene, alpha- & beta-carotene, alpha- & gamma-tocopherol, C-reactive protein, triglycerides, total, LDL, and HDL cholesterol.

For the first subsample (BA23) consisting of 2098 samples, DNA methylation levels were measured using the Illumina Infinium HumanMethylation450 BeadChip at the HudsonAlpha Institute of Biotechnology. This platform uses bisulfite conversion to quantify methylation levels at 485,577 specific CpG sites genome-wide. Samples were prepared according to the standard Illumina protocol, and β methylation values were calculated from the intensity ratio between methylated and total (methylated and unmethylated) probe fluorescence intensities. Methylation data was processed as described in [38]. In order to test the quality of these array measurements, we perform correlation measures with duplicates within this dataset and with a "gold" standard which is an average of many samples previously collected. Correlation between duplicates and with the gold standard were high (r>0.9), indicative of high quality measurements.

The second WHI data set is described in the following.

### WHI-EMPC description

The Women's Health Initiative – Epigenetic Mechanisms of PM-Mediated CVD (WHI-EMPC, AS315) is an ancillary study of epigenetic mechanisms underlying associations between ambient particulate matter (PM) air pollution and cardiovascular disease (CVD) in the Women's Health Initiative clinical trials (CT) cohort. The WHI-EMPC study population is a stratified, random sample of 2,200 WHI CT participants who were examined between 1993 and 2001; had available buffy coat, core analytes, electrocardiograms, and ambient concentrations of PM; but were not taking anti-arrhythmic medications at the time. As such, WHI-EMPC is representative of the larger, multiethnic WHI CT population from which it was sampled: n = 68,132 participants aged 50-79 years who were randomized to hormone therapy, calcium/vitamin D supplementation, and/or dietary modification in 40 U.S. clinical centers at the baseline exam (1993-1998) and re-examined in the fasting state one, three, six, and nine years later [93].

Illumina Infinium HumanMethylation450 BeadChip data from the Northwestern University Genomics Core Facility for WHI-EMPC participants sampled in stages 1a (800 participants), 1b (1200 participants), and 2 (200 participants x 2 samples each) was quality controlled and batch adjusted. Batch adjustment involved applying empirical Bayes methods of adjusting for stage and plate as implemented in ComBat [94].

### Dietary assessment in the Invecchiare nel Chianti (InCHIANTI)

The InCHIANTI Study is a population-based prospective cohort study of residents ages 30 or older from two areas in the Chianti region of Tuscany, Italy. Data on demographic and lifestyle factors such as smoking, years of education, BMI, and physical activity were collected during the baseline interview. Physical activity in the previous year was categorized as sedentary or active. Smoking was categorized into current smoker versus former or non-smokers (Table 1). In the InCHIANTI study, dietary intake for the past year was assessed using a 236 item food frequency questionnaire (FFQ) for the European Prospective Investigation on Cancer and nutrition (EPIC) study, previously validated in the InCHIANTI population [95]. The FFQ was administered by a trained interviewer and collected information on how frequently (weekly, monthly, yearly) each specific food was generally consumed. Participants were asked to specify the size of the portion usually consumed, in comparison to a range of portion that are shown in colored photographs. Nutrient data for specific foods were obtained from the Food Composition Database for Epidemiological Studies in Italy [96]. Dietary information was judged as unreliable and excluded from further analysis if reported energy intakes were <600 kcal/day or >4,000 kcal/day and >4,200 kcal/day in women and men, respectively.

### Blood biomarkers and DNA methylation in the InCHIANTI

Sampling and data collection procedures have been described elsewhere [97]. Briefly, participants were enrolled between 1998 and 2000 and were examined at three-year intervals. Serum samples obtained from blood collected in evacuated tubes without anti-coagulant were centrifuged at 2000g for 10 min, and stored at -80°C for measurement of glucose, total, LDL, and HDL, cholesterol, triglycerides, CRP, and creatinine. DNA methylation was assayed using the Illumina Infinium HumanMethylation450 platform for n=407 participants with sufficient DNA at both baseline (years 1998-2000) and year 9 follow-up visits (2007-2009).

### Assessment of metabolic syndrome

Metabolic syndrome status was assessed using the ATPIII NCEP 2004 criteria defined by the presence of 3 or more of the following characteristics: waist circumference >88cm (if male, >102cm), systolic blood pressure >130mmHg or diastolic blood pressure >85mmHg, fasting plasma glucose >100mg/dL, HDL cholesterol <50mg/dL (if male, <40mg/dL), and triglycerides >150mg/dL. In regression models, we use total number of metabolic syndrome characteristics as an ordinal variable, ranging from 0 to 5.

### Statistical analyses

#### Dietary analysis

Biweight midcorrelation, an outlier-robust correlation measure, was used to assess marginal linear relationships between epigenetic aging measures and dietary, cardiometabolic, and socioeconomic factors. To adjust for possible socioeconomic and lifestyle confounders, we fit ethnically-stratified multivariable linear models adjusting for education, exercise, BMI, and current drinker and smoker status. We used Stouffer's method to infer the meta-analytic significance of each variable over the different ethnic strata using the square-root of the sample size as the Z-score weighting factor. Specifically for the WHI, the age acceleration measures were adjusted for differences in originating dataset and within the InCHIANTI the measures were adjusted for sex. Models including regression on biomarkers, and number of metabolic syndrome symptoms were not stratified by ethnicity due to lack of coverage for biomarker profiling. Models were designed based on common prior knowledge and in cases where there was co-linearity between confounding variables, choice for adjustment was selected based on variable commonality in order to improve comparability with other studies, e.g. BMI was chosen over WHR because BMI is more commonly measured and reported. Variables with skewness >1 were log transformed (possibly adding +1 to avoid forming the logarithm of zero). Mean carotenoids was computed as the mean across standardized measures of lycopene, log2(alpha-carotene), log2(beta-carotene), log2(lutein + zeaxanthin), and log2(beta-cryptoxanthin). Repeat measurements on the same individuals were omitted from the analysis.

#### Metformin analysis

Prescription medication use was collected at baseline for women in both the clinical trial and the observational study. Follow-up recording of new medication was done at years 1, 3, 6, and 9 for women enrolled in clinical trial and at year 3 for those enrolled in the observational study. At each of these waves, participants were asked to bring in their prescription medication bottles and interviewers recorded the National Drug Code (NDC), the product name, and generic name for each drug. In our analysis, those who took prescription drugs that fell under the Therapeutic Class Codes (TCCODE), 272500 or 279970 were coded as taking Metformin. TCCODE 272500 covered the following drugs: Metformin HCL, Metformin ER, Fortamet, Glucophage XR, Glucophage, and Riomet; all of which were Metformin HCL tablets. TCCODE 279970 covered the following drugs: Glucovance, Glyburide/Metformin HCL, and Metaglip; the first two of which were Glyburide and Metformin HCL combination tablets, whereas the later is a Glipizide Metformin HCL combination tablet. In our study, approximately 12% (n=489) of women were reported as using Metformin at some time during the study. Women were also asked the age at which they started using prescribed medication. On average, women in our study began metformin use at age 65, with the youngest and oldest ages of initiation reported as 41 and 85, respectively.

We utilized the reported age of Metformin initiation to estimate whether women were using metformin at the time of blood draw (when DNAm age was measured), and if so, how long they had been on the drug. For those women who started using Metformin after the date of blood draw, we were also able to calculate the time between blood draw and starting on Metformin. Based on this, we classified women in to three categories—“No Metformin” (those who never used Metformin), “Metformin Now” (those on Metformin at the time of blood draw), and “Metformin Future” (those who began Metformin after blood draw). In our sample, 4,073 women were in the No Metformin group, 139 were in the Metformin Now group, and 350 were in the Metformin Future group.

Kruskal Wallis tests were used to compare the ages at blood draw, education levels, race/ethnicity, fasting glucose levels, fasting insulin levels, and degree of insulin resistance (measured using the Homeostatic Model Assessment of Insulin Resistance--HOMA_IR). Biweight midcorrelations were used to examine whether these characteristics differed as a function of the timing of Metformin initiation among those on Metformin at some point during the study. Next Kruskal Wallis tests and biweight midcorrelations were used to examine whether Metformin group classification and/or the timing of Metformin use was associated with IEAA or EEAA. For these models, both IEAA and EEAA were adjusted for age at blood draw, education, and race/ethnicity. These models were run using the full sample, and stratifying by age at blood draw (<65 years vs. 65+ years), race/ethnicity, and fasting glucose levels (<126 mg/dL vs. 126+ mg/dL). Next, a two group comparison was made for the full sample and stratifying by race/ethnicity between a) 47 women in the Metformin Now group for whom Metformin was potentially effective (fasting glucose < 140 mg/dL), and b) the 350 women in the Metformin Future group. Finally, there were 308 women who 1) had IEAA and EEAA measured during at least two separate WHI visits and 2) were not on Metformin at the time of their first blood draw. Of these women, 11 started Metformin in between their two blood draws. Based on this, we used linear models to compare the change in IEAA and EEAA between women who started Metformin between their first and second IEAA and EEAA measure and those who did not. Models were adjusted for age at first blood draw, age at second blood draw, race/ethnicity, education, glucose levels at first blood draw, and either IEAA or EEAA at first blood draw.

## SUPPLEMENTARY INFORMATION


