# Supplementary material for: Epigenetic clock analysis of diet, exercise, education, and lifestyle factors
Source: Aging (Albany NY). 2017 Feb 14;9(2):419–37. doi: 10.18632/aging.101168 (PMC5361673; doi:10.18632/aging.101168)
Supplement: Supplementary file 1 [file aging-09-419-s001.pdf]

SUPPLEMENTARY INFORMATION

Selection of participants from the Women's Health Initiative BA23 dataset

Participants included individuals from the Women's Health Initiative (WHI), a national study that began in 1993 and enrolled postmenopausal women between the ages of 50-79 years into either randomized clinical trials (RCTs) or into an observational study; in our sample, 1,657 of these individuals were enrolled in at least one of the RCTs and the remaining 441 were enrolled in the observational study. For this analysis, women were selected from one of two large WHI sub-cohorts that had previously undergone genotyping as well as profiling for cardiovascular disease related blood biomarkers through two core WHI ancillary studies (Supplementary Figure 10).

The first cohort is known as the WHI SNP Health Association Resource (SHARe) cohort of minorities that includes >8000 African American women and >3500 Hispanic women. These women were genotyped through the WHI core study M5-SHARe (www.whi.org/researchers/data/WHIStudies/StudySites/M5) and underwent baseline blood biomarker profiling through WHI Core study W54-SHARe (www.whi.org/researchers/data/WHIStudies/StudySites/M5). The second cohort consists of a combination of European Americans from the two Hormonal Therapy (HT) trials selected for GWAS and biomarkers in core studies W58 (www.whi.org/researchers/data/WHI Studies/StudySites/W58) and W63 (www.whi.org/researchers/data/ HIStudies/StudySites/ W63).

While none of the participants of our study suffered from coronary heart disease at baseline, roughly half of the samples developed CHD at a later point. From the M5-SHARe and W54-SHARe cohorts, two sample sets were formed. The first (sample set 1) is a sample set of 637 CHD cases and 631 non-CHD cases as of Sept 30, 2010. The second sample set (sample set 2) is a non-overlapping sample of 432 cases of coronary heart disease and 472 non-cases as of September 17, 2012. We acknowledge a potential for selection bias using the above-described sampling scheme in WHI but suspect if such bias is present it is minimal. First, some selection bias is introduced by restricting our methylation profiling at baseline to women with GWAS & biomarker data from baseline as well, given the requirement that these participants must have signed the WHI supplemental consent for broad sharing of genetic data in 2005. However, we believe that selection bias at this stage is minimized by the inclusion of participants who died between the time of the start of the WHI study and the time of supplemental consent in 2005, which resulted in the exclusion of only ~6-8% of all WHI participants. Nevertheless, participants unable or unwilling to sign consent in 2005 may not represent a random subset of all participants who survived to 2005. Second, some selection bias may also occur if similar gross differences exist in the characteristics of participants who consented to be followed in the two WHI extension studies beginning in 2005 and 2010 compared to non-participants at each stage. We believe these selection biases, if present, have minimal influence on our effect estimates.

A

		WHI BA23 dataset																							
		Adjusted for ethnicity						Caucasian						African American						Hispanic					
				EEAA		IEAA				EEAA		IEAA				EEAA		IEAA				EEAA		IEAA	
		n	μ	bicor	p	bicor	p	n	μ	bicor	p	bicor	p	n	μ	bicor	p	bicor	p	n	μ	bicor	p	bicor	p
Diet	log2(Total energy)	2086	10.51	-0.02	0.33	0.00	0.87	992	10.58	-0.04	0.17	0.00	0.90	666	10.46	0.03	0.40	-0.01	0.85	428	10.45	-0.06	0.20	0.01	0.90
	Carbohydrate	2086	49.40	-0.01	0.59	0.04	0.05	992	49.45	0.01	0.81	0.05	0.11	666	49.03	-0.03	0.47	0.02	0.55	428	49.89	-0.02	0.61	0.06	0.19
	Protein	2086	16.54	-0.02	0.31	-0.03	0.24	992	16.89	-0.02	0.48	0.01	0.71	666	16.10	-0.02	0.70	-0.08	0.03	428	16.40	-0.02	0.65	-0.01	0.87
	Fat	2086	34.20	0.03	0.20	-0.02	0.45	992	33.23	0.01	0.71	-0.02	0.52	666	35.65	0.02	0.66	0.00	0.96	428	34.18	0.09	0.07	-0.05	0.35
	log2(1+Red meat)	2086	0.74	0.02	0.41	0.00	0.95	992	0.75	-0.02	0.51	-0.01	0.70	666	0.73	0.07	0.06	0.00	0.94	428	0.72	0.01	0.87	0.02	0.67
	log2(1+Poultry)	2086	0.46	-0.04	0.06	-0.05	0.02	992	0.41	-0.05	0.15	-0.05	0.09	666	0.52	-0.02	0.69	-0.08	0.04	428	0.47	-0.07	0.17	0.01	0.89
	log2(1+Fish)	2086	0.31	-0.08	6E-4	-0.02	0.34	992	0.29	-0.07	0.04	0.02	0.52	666	0.37	-0.06	0.11	-0.09	0.03	428	0.25	-0.11	0.02	0.00	0.94
	log2(1+Dairy)	2086	1.25	-0.02	0.26	-0.02	0.43	992	1.42	-0.03	0.30	-0.01	0.72	666	1.02	0.01	0.84	-0.02	0.53	428	1.23	-0.06	0.24	-0.02	0.63
	log2(1+Whole grains)	2086	1.03	-0.01	0.58	0.01	0.76	992	1.00	-0.04	0.16	0.00	0.97	666	1.03	0.04	0.36	0.00	0.97	428	1.07	-0.02	0.63	0.03	0.52
	log2(1+Nuts)	2086	0.18	0.01	0.58	0.03	0.13	992	0.20	0.03	0.31	0.05	0.09	666	0.20	0.01	0.88	0.02	0.58	428	0.11	0.02	0.71	0.05	0.27
	log2(Fruits)	2086	0.33	-0.03	0.20	0.02	0.40	992	0.47	-0.04	0.19	0.02	0.63	666	0.28	0.02	0.57	0.02	0.64	428	0.11	-0.07	0.12	0.02	0.65
	log2(Vegetables)	2086	0.60	-0.05	0.03	0.02	0.34	992	0.93	-0.06	0.08	0.02	0.59	666	0.44	0.01	0.85	0.01	0.89	428	0.10	-0.12	0.02	0.04	0.37
Blood nutrients	Retinol	224	0.59	-0.09	0.18	0.00	0.99	74	0.65	0.05	0.70	0.21	0.08	85	0.57	-0.13	0.24	-0.10	0.38	65	0.56	-0.18	0.15	-0.15	0.24
	Mean carotenoids	224	0.03	-0.19	4E-3	-0.03	0.63	74	0.08	-0.21	0.07	-0.07	0.55	85	-0.02	-0.13	0.22	-0.04	0.75	65	0.03	-0.25	0.04	-0.01	0.95
	Lycopene	224	0.42	-0.12	0.07	-0.03	0.66	74	0.45	-0.11	0.36	0.07	0.56	85	0.37	-0.15	0.18	-0.09	0.40	65	0.44	-0.17	0.17	-0.09	0.49
	log2(alpha-Carotene)	224	-4.28	-0.16	0.02	0.01	0.88	74	-4.07	-0.14	0.24	-0.04	0.74	85	-4.60	-0.11	0.31	0.02	0.85	65	-4.11	-0.22	0.07	0.05	0.70
	log2(beta-Carotene)	224	-2.09	-0.19	4E-3	-0.08	0.26	74	-1.94	-0.13	0.28	-0.03	0.77	85	-2.02	-0.16	0.14	-0.08	0.45	65	-2.35	-0.27	0.03	-0.11	0.38
	log2(Lutein+Zeaxanthin)	224	-2.36	-0.13	0.06	-0.06	0.38	74	-2.44	-0.08	0.52	-0.05	0.70	85	-2.22	-0.13	0.23	-0.16	0.15	65	-2.45	-0.17	0.17	0.08	0.54
	log2(beta-Cryptoxanthin)	224	-3.82	-0.04	0.52	0.03	0.67	74	-3.94	-0.15	0.19	-0.01	0.96	85	-3.82	0.09	0.43	0.08	0.48	65	-3.66	-0.06	0.66	0.02	0.88
	log2(alpha-Tocopherol)	224	3.90	-0.04	0.54	0.00	1.00	74	4.03	-0.10	0.40	0.03	0.78	85	3.81	0.04	0.75	-0.12	0.27	65	3.86	-0.05	0.69	0.13	0.30
	log2(gamma-Tocopherol)	224	0.87	0.05	0.48	0.12	0.07	74	0.87	0.00	0.98	0.07	0.54	85	0.96	0.15	0.17	0.19	0.08	65	0.74	-0.01	0.93	0.08	0.55
Measurements	log2(C-reactive protein)	2073	1.58	0.11	3E-7	0.07	2E-3	991	1.26	0.12	3E-4	0.07	0.04	661	1.90	0.07	0.06	0.08	0.03	421	1.81	0.19	1E-4	0.05	0.33
	log2(Insulin)	2051	5.82	0.09	3E-5	0.06	3E-3	970	5.65	0.12	2E-4	0.06	0.05	656	6.05	0.06	0.13	0.09	0.02	425	5.86	0.09	0.06	0.02	0.65
	log2(Glucose)	2091	6.70	0.04	0.08	0.06	0.01	995	6.67	0.07	0.02	0.05	0.15	666	6.74	0.02	0.62	0.07	0.07	430	6.70	0.00	0.93	0.07	0.17
	log2(Triglyceride)	2091	7.02	0.09	2E-5	0.08	2E-4	995	7.12	0.10	3E-3	0.08	0.02	666	6.76	0.08	0.04	0.09	0.02	430	7.21	0.11	0.02	0.08	0.09
	Total cholesterol	2091	233.03	0.04	0.06	0.03	0.16	995	236.31	0.08	0.01	0.04	0.24	666	231.75	0.02	0.66	0.01	0.79	430	227.37	0.00	0.98	0.04	0.37
	LDL cholesterol	2057	151.23	0.03	0.18	0.01	0.58	976	153.81	0.07	0.02	0.02	0.54	659	152.79	0.01	0.82	0.00	0.92	422	142.83	-0.03	0.50	0.02	0.74
	HDL cholesterol	2091	52.17	-0.06	4E-3	-0.04	0.09	995	51.01	-0.06	0.05	-0.06	0.06	666	54.71	-0.06	0.14	-0.02	0.62	430	50.91	-0.07	0.14	-0.02	0.64
	log2(Creatinine)	2041	-0.42	0.01	0.59	-0.01	0.67	973	-0.45	0.03	0.43	-0.02	0.62	657	-0.33	0.05	0.19	0.03	0.40	411	-0.50	-0.07	0.14	-0.07	0.19
	Systolic blood pressure	2093	132.02	0.05	0.01	0.02	0.26	995	131.92	0.10	2E-3	0.01	0.85	668	134.05	0.03	0.37	0.07	0.08	430	129.06	-0.02	0.63	-0.01	0.88
	Diastolic blood pressure	2093	76.41	0.03	0.24	0.03	0.13	995	75.21	0.06	0.05	0.02	0.49	668	78.71	0.00	0.98	0.08	0.05	430	75.58	-0.03	0.59	-0.01	0.78
	log2(Waist / hip ratio)	2093	-0.27	0.09	8E-5	0.04	0.10	995	-0.28	0.09	0.01	0.06	0.06	668	-0.27	0.06	0.13	-0.01	0.77	430	-0.28	0.13	0.01	0.06	0.23
	BMI	2093	29.85	0.08	6E-4	0.07	2E-3	995	28.83	0.10	1E-3	0.10	2E-3	668	31.71	0.05	0.20	0.06	0.13	430	29.32	0.06	0.20	0.01	0.81
Socio-behavioral	Education	2071	6.66	-0.10	2E-6	0.00	0.83	989	6.86	-0.12	2E-4	0.02	0.43	660	6.85	-0.11	0.01	-0.03	0.40	422	5.90	-0.06	0.20	0.02	0.61
	Income	2019	3.54	-0.08	3E-4	-0.01	0.51	964	3.67	-0.06	0.06	0.02	0.64	642	3.47	-0.08	0.04	-0.06	0.11	413	3.32	-0.11	0.02	0.01	0.80
	log2(1+Exercise)	2090	2.49	-0.08	5E-4	-0.03	0.20	994	2.67	-0.08	0.01	0.01	0.70	667	2.30	-0.04	0.26	-0.11	4E-3	429	2.37	-0.12	0.01	0.02	0.67
	Current smoker	2090	0.14	0.00	0.93	0.00	0.88	994	0.13	-0.03	0.37	0.01	0.80	667	0.16	0.07	0.07	0.02	0.60	429	0.14	-0.01	0.77	0.05	0.27
	log2(1+Alcohol)	2086	1.09	-0.05	0.02	-0.01	0.52	992	1.44	-0.06	0.07	-0.02	0.48	666	0.73	-0.01	0.72	0.01	0.79	428	0.86	0.00	0.92	0.01	0.82

B

		WHI AS315 dataset																							
		Adjusted for ethnicity						Caucasian						African American						Hispanic					
				EEAA		IEAA				EEAA		IEAA				EEAA		IEAA				EEAA		IEAA	
		n	μ	bicor	p	bicor	p	n	μ	bicor	p	bicor	p	n	μ	bicor	p	bicor	p	n	μ	bicor	p	bicor	p
Diet	log2(Total energy)	1601	10.56	-0.02	0.49	0.01	0.77	828	10.62	-0.03	0.37	0.05	0.13	405	10.49	0.04	0.45	0.03	0.52	205	10.58	-0.03	0.71	-0.14	0.05
	Carbohydrate	1601	48.50	0.02	0.47	-0.03	0.30	828	48.09	0.03	0.34	-0.04	0.24	405	48.67	0.03	0.49	0.01	0.88	205	48.34	-0.03	0.68	0.06	0.37
	Protein	1601	16.45	-0.03	0.27	-0.02	0.36	828	16.73	-0.06	0.07	-0.04	0.21	405	15.87	-0.05	0.29	-0.01	0.82	205	16.43	0.08	0.25	-0.01	0.87
	Fat	1601	35.28	0.01	0.58	0.03	0.23	828	34.98	0.03	0.37	0.06	0.11	405	36.32	-0.04	0.47	-0.03	0.57	205	35.65	0.05	0.44	-0.04	0.57
	log2(1+Red meat)	1601	0.77	0.02	0.42	0.07	0.01	828	0.79	0.02	0.59	0.10	3E-3	405	0.76	0.03	0.54	0.09	0.06	205	0.82	0.05	0.46	-0.09	0.20
	log2(1+Poultry)	1601	0.45	-0.02	0.43	-0.04	0.10	828	0.43	-0.05	0.19	-0.02	0.59	405	0.50	0.03	0.54	0.00	0.94	205	0.49	0.01	0.90	-0.16	0.02
	log2(1+Fish)	1601	0.32	-0.06	0.01	-0.01	0.59	828	0.31	-0.07	0.04	0.03	0.46	405	0.36	-0.05	0.27	-0.09	0.09	205	0.26	-0.03	0.72	-0.08	0.24
	log2(1+Dairy)	1601	1.26	-0.01	0.82	0.02	0.37	828	1.42	-0.04	0.27	0.04	0.27	405	1.00	0.05	0.27	0.08	0.11	205	1.30	0.06	0.37	-0.04	0.59
	log2(1+Whole grains)	1601	-0.17	-0.04	0.13	-0.02	0.45	828	-0.13	-0.04	0.27	0.01	0.79	405	-0.21	-0.06	0.26	-0.02	0.65	205	0.02	0.06	0.40	-0.11	0.13
	log2(1+Nuts)	1601	0.21	-0.05	0.06	-0.01	0.57	828	0.23	-0.03	0.36	0.00	0.93	405	0.21	-0.10	0.05	0.00	0.99	205	0.13	0.07	0.35	0.02	0.75
	log2(Fruits)	1601	0.31	-0.04	0.10	-0.03	0.17	828	0.37	-0.08	0.02	-0.03	0.36	405	0.34	0.01	0.84	0.03	0.53	205	0.10	0.00	0.99	-0.09	0.21
	log2(Vegetables)	1601	0.64	-0.03	0.22	-0.03	0.28	828	0.81	0.00	0.98	0.00	0.95	405	0.54	-0.02	0.63	-0.01	0.81	205	0.15	-0.09	0.18	-0.12	0.08
Blood nutrients	Retinol	2044	0.59	0.00	0.98	0.02	0.43	1040	0.62	0.04	0.20	0.03	0.31	511	0.56	-0.02	0.60	0.03	0.49	281	0.54	-0.06	0.28	-0.01	0.85
	Mean carotenoids	2043	0.01	-0.12	7E-8	-0.06	4E-3	1040	-0.03	-0.14	5E-6	-0.09	4E-3	510	-0.02	-0.12	5E-3	-0.06	0.21	281	0.05	-0.02	0.76	-0.04	0.54
	Lycopene	2044	0.39	-0.02	0.37	-0.01	0.52	1040	0.40	0.00	0.97	-0.02	0.55	511	0.39	-0.03	0.44	-0.05	0.31	281	0.40	-0.02	0.80	0.00	0.94
	log2(alpha-Carotene)	2044	-4.22	-0.11	1E-6	-0.05	0.03	1040	-4.13	-0.11	2E-4	-0.06	0.07	511	-4.53	-0.13	4E-3	-0.05	0.23	281	-4.10	-0.06	0.35	-0.06	0.30
	log2(beta-Carotene)	2043	-2.19	-0.10	8E-6	-0.05	0.01	1040	-2.22	-0.11	6E-4	-0.06	0.04	510	-2.17	-0.12	0.01	-0.06	0.21	281	-2.36	0.01	0.90	0.00	0.95
	log2(Lutein+Zeaxanthin)	2044	-2.39	-0.09	8E-5	-0.03	0.15	1040	-2.47	-0.10	8E-4	-0.05	0.09	511	-2.27	-0.08	0.06	0.00	0.97	281	-2.46	-0.06	0.32	-0.09	0.13
	log2(beta-Cryptoxanthin)	2044	-3.74	-0.11	2E-7	-0.07	9E-4	1040	-3.91	-0.17	5E-8	-0.11	4E-4	511	-3.78	-0.11	0.01	-0.05	0.30	281	-3.41	0.05	0.38	0.02	0.75
	log2(alpha-Tocopherol)	2044	3.94	-0.06	0.01	-0.04	0.06	1040	4.01	-0.10	2E-3	-0.10	2E-3	511	3.74	-0.02	0.64	0.10	0.03	281	3.90	-0.05	0.38	-0.07	0.23
	log2(gamma-Tocopherol)	2044	0.66	0.10	1E-5	0.07	8E-4	1040	0.58	0.13	5E-5	0.11	3E-4	511	0.99	0.08	0.08	0.00	0.99	281	0.67	0.06	0.30	-0.01	0.83
Measurements	log2(C-reactive protein)	736	1.44	0.16	9E-6	0.09	0.01	342	1.14	0.18	1E-3	0.18	8E-4	251	1.88	0.16	0.01	-0.01	0.88	113	1.27	0.10	0.30	0.08	0.38
	log2(Insulin)	1992	5.79	0.14	9E-10	0.07	3E-3	1009	5.64	0.11	7E-4	0.06	0.08	506	6.01	0.18	6E-5	0.08	0.08	277	5.96	0.15	0.01	0.02	0.69
	log2(Glucose)	2054	6.63	0.08	4E-4	0.05	0.02	1042	6.60	0.03	0.28	0.06	0.05	513	6.68	0.11	0.01	0.02	0.72	285	6.63	0.18	2E-3	0.12	0.04
	log2(Triglyceride)	2058	7.07	0.05	0.02	0.02	0.36	1046	7.15	0.01	0.77	0.01	0.70	513	6.75	0.09	0.05	0.06	0.16	285	7.20	0.04	0.47	-0.06	0.33
	Total cholesterol	2058	221.49	-0.02	0.27	0.03	0.15	1046	224.23	-0.01	0.87	0.00	0.96	513	220.40	-0.05	0.29	0.07	0.10	285	215.90	0.00	0.99	0.06	0.31
	LDL cholesterol	2028	134.34	0.00	0.87	0.05	0.04	1027	134.76	0.03	0.40	0.02	0.58	512	139.70	0.00	0.97	0.07	0.10	280	129.53	0.01	0.84	0.13	0.03
	HDL cholesterol	2055	57.60	-0.12	2E-8	-0.03	0.13	1043	58.39	-0.09	2E-3	-0.03	0.29	513	57.38	-0.20	5E-6	0.01	0.89	285	54.48	-0.10	0.09	-0.09	0.11
	log2(Creatinine)	707	-0.43	0.06	0.12	0.06	0.11	326	-0.47	0.09	0.10	0.06	0.30	243	-0.32	0.05	0.48	0.06	0.35	109	-0.52	0.05	0.58	0.04	0.69
	Systolic blood pressure	2072	128.27	0.10	8E-6	0.06	0.01	1051	127.42	0.07	0.02	0.04	0.22	517	131.31	0.16	3E-4	0.14	1E-3	286	126.99	0.09	0.13	0.03	0.61
	Diastolic blood pressure	2072	75.29	0.06	0.01	0.06	0.01	1051	74.52	0.05	0.10	0.06	0.04	517	77.30	0.11	0.01	0.08	0.08	286	74.75	0.08	0.20	0.04	0.47
	log2(Waist / hip ratio)	2072	-0.29	0.09	2E-5	0.05	0.02	1051	-0.30	0.08	0.01	0.04	0.18	517	-0.28	0.12	0.01	0.08	0.07	286	-0.28	0.07	0.23	0.03	0.63
	BMI	2072	29.51	0.10	2E-6	0.08	2E-4	1051	28.88	0.07	0.02	0.06	0.05	517	31.55	0.15	4E-4	0.09	0.04	286	29.70	0.10	0.10	0.02	0.71
Socio-behavioral	Education	2059	6.95	-0.09	2E-5	-0.05	0.02	1047	7.08	-0.09	3E-3	-0.05	0.15	512	7.02	-0.07	0.09	-0.12	0.01	283	6.03	-0.17	4E-3	-0.03	0.67
	Income	2022	3.92	-0.05	0.03	0.01	0.75	1025	4.08	-0.05	0.09	0.01	0.75	504	3.68	0.03	0.53	0.01	0.84	277	3.42	-0.10	0.11	-0.07	0.26
	log2(1+Exercise)	2052	2.57	-0.06	0.01	-0.05	0.02	1046	2.74	-0.08	0.01	-0.07	0.03	509	2.21	-0.06	0.17	-0.01	0.74	283	2.28	0.06	0.33	0.02	0.76
	Current smoker	2052	0.10	0.00	0.99	-0.01	0.69	1046	0.10	0.08	0.01	0.01	0.64	509	0.12	0.00	0.97	-0.04	0.41	283	0.10	0.00	0.95	0.02	0.77
	log2(1+Alcohol)	1601	1.11	-0.10	1E-4	-0.03	0.28	828	1.43	-0.12	4E-4	-0.04	0.27	405	0.69	0.00	0.96	-0.05	0.32	205	0.91	-0.11	0.13	-0.06	0.43

Supplementary Figure 1. Related to Figure 1. Stratified correlation analysis for IEAA and EEAA in the WHI. There are two main tables: the top original WHI sample, and the bottom validation sample. The correlations (bicor, biweight midcorrelation) for the two measures of epigenetic age acceleration are colored according to their magnitude, with positive correlations in red, negative correlations in blue, and statistical significance (p-values) in green. Smaller test sample sizes (n) are highlighted in orange, and sample means (μ) are listed. Stratified ethnic groups are displayed as separate groups of columns with the first column showing them in an adjusted aggregate. Blood biomarkers were measured from fasting plasma collected at baseline. Food groups and nutrients are inclusive, including all types and all preparation methods, e.g. folic acid includes synthetic and natural, dairy includes cheese and all types of milk, etc. Minor ethnic strata (Asians, American Indians, and other) in the validation sample are excluded because of small sample are excluded for for clarity.

Supplementary Table 1. Linear models of IEAA and EEAA in WHI metformin users with repeat measurements.

	IEAA		EEAA	
	β Coefficient	P-Value	β Coefficient	P-Value
Started Metformin	-0.79	0.456	-0.003	0.998
IEAA/EEAA (First Blood Draw)	-0.33	2.88e-13	-0.35	8.92e-15
Age (First Blood Draw)	-0.55	1.73e-08	-0.96	4.67e-15
Age (Second Blood Draw)	0.50	6.02e-07	0.98	1.21e-14
Glucose (First Blood Draw)	0.02	0.132	0.03	0.048
Glucose (First Blood Draw)	0.01	0.657	-0.03	0.056
Education	0.27	0.004	-0.02	0.89
Race/Ethnicity (Reference=Non-Hispanic White)				
American Indian	-1.03	0.577	-1.31	0.555
Asian	-1.10	0.450	-3.54	0.044
Non-Hispanic Black	-2.04	0.166	-2.45	0.165
Hispanic	-0.63	0.658	-2.88	0.093
Other	1.32	0.614	-3.60	0.254

Table shows the beta-coefficients and corresponding p-values for linear models of IEAA and EEAA in the middle and right columns, respectively. The left column denotes the regressors in the models.

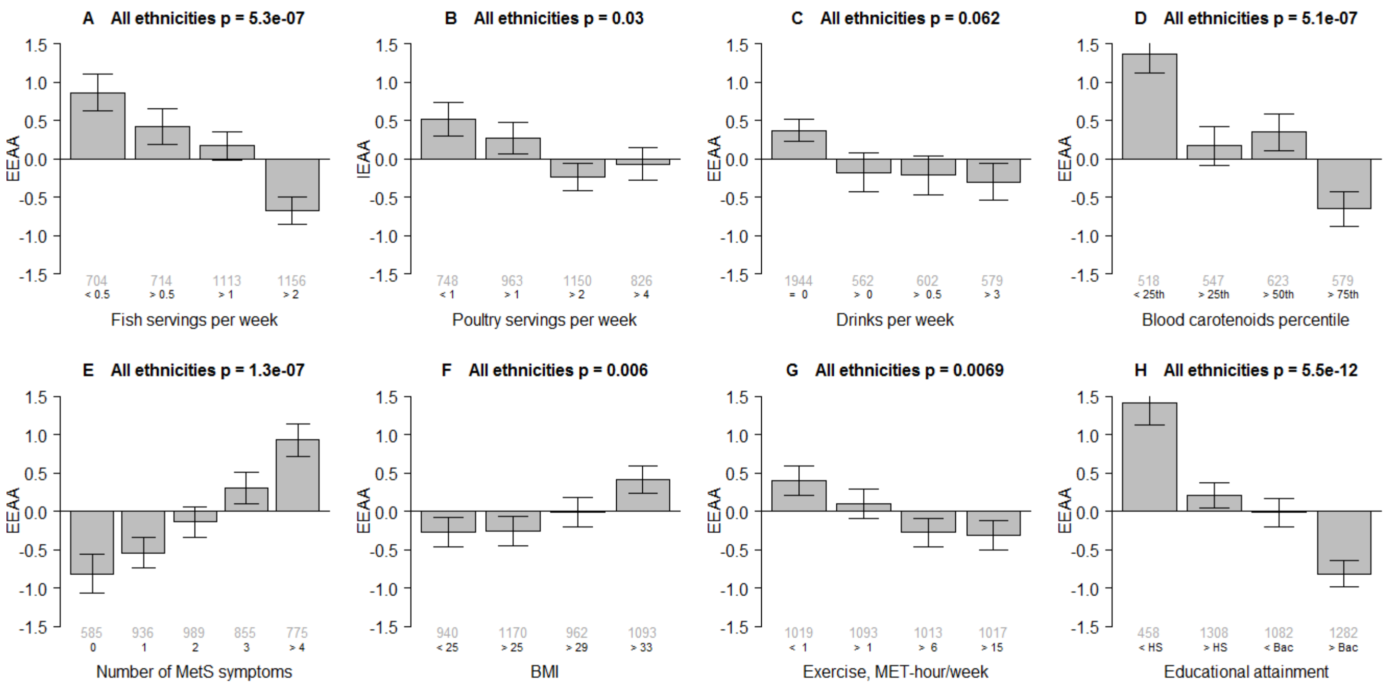

Supplementary Figure 2. EEAA among different levels of select dietary & lifestyle habits and measurements in the WHI. Panels **A**-**H** show bar plots visualizing the EEAA among stratified levels of fish, poultry (IEAA in this case), alcohol intake, blood carotenoid levels, number of metabolic syndrome symptoms, BMI, exercise, and education. Cut points roughly correspond roughly to quartiles except with number of MetS symptoms and alcohol intake which were selected for evenly-sized strata as much as possible. The sample sizes for each stratum are shown in grey beneath each bar. P-values for differences between strata are listed above each bar plot. Exercise is measured in units of metabolic equivalent hours per week and education uses high school diploma, some college, and bachelor's degree as cut points.

Pairwise correlations Adjusted for ethnicity and dataset		Diet												Blood nutrients									Measurements												Socio-behavioral					AA	
		log2(Total energy)	Carbohydrate	Protein	Fat	log2(1+Red meat)	log2(1+Poultry)	log2(1+Fish)	log2(1+Dairy)	log2(1+Whole grains)	log2(1+Nuts)	log2(Fruits)	log2(Vegetables)	Retinol	Mean carotenoids	Lycopene	log2(alpha-Carotene)	log2(beta-Carotene)	log2(Lutein+Zeaxanthin)	log2(beta-Cryptoxanthin)	log2(alpha-Tocopherol)	log2(gamma-Tocopherol)	log2(C-reactive protein)	log2(Insulin)	log2(Glucose)	log2(Triglyceride)	Total cholesterol	LDL cholesterol	HDL cholesterol	log2(Creatinine)	Systolic blood pressure	Diastolic blood pressure	log2(Waist / hip ratio)	BMI	Education	Income	log2(1+Exercise)	Current smoker	log2(1+Alcohol)	EEAA	IEAA
Diet	log2(Total energy)		-22	-2	24	62	48	35	63	52	25	25	32	-3	0	2	0	-3	-2	2	-3	1	6	7	3	2	-6	-6	-3	-1	1	5	6	11	7	4	-1	0	7	-2	0
	Carbohydrate	-22		-21	-83	-52	-23	-12	-4	15	-7	42	14	3	16	-3	19	17	8	19	10	-14	-14	-13	-9	2	1	2	-4	5	1	-4	-8	-15	6	-1	13	-1	-14	0	2
	Protein	-2	-21		-11	24	40	26	22	5	-3	-1	17	7	6	2	6	6	7	-1	7	-6	4	3	5	1	-2	-2	-1	-2	-2	-1	1	9	5	3	9	1	-2	-3	-2
	Fat	24	-83	-11		45	9	-1	1	-11	12	-39	-20	-9	-21	0	-22	-20	-15	-20	-12	17	15	16	8	2	-3	-3	-2	-3	1	4	9	18	-11	-5	-21	1	-7	2	0
	log2(1+Red meat)	62	-52	24	45		38	18	29	19	13	-6	8	1	-16	8	-19	-18	-15	-13	-8	13	13	17	9	7	-5	-5	-8	-1	2	3	12	19	-7	1	-12	2	5	2	3
	log2(1+Poultry)	48	-23	40	9	38		32	27	27	10	11	24	0	3	3	4	2	4	2	1	-3	6	7	4	1	-2	-3	0	-2	-1	3	3	11	7	9	1	1	9	-3	-5
	log2(1+Fish)	35	-12	26	-1	18	32		23	19	11	16	28	4	8	4	7	6	10	3	8	-11	0	-1	1	-3	2	1	5	-2	-2	5	-1	6	14	14	9	1	19	-7	-2
	log2(1+Dairy)	63	-4	22	1	29	27	23		37	21	21	20	2	2	-1	7	2	-2	3	1	-6	1	2	1	-1	-5	-5	0	0	0	1	2	6	10	3	6	1	5	-2	0
	log2(1+Whole grains)	52	15	5	-11	19	27	19	37		20	21	28	2	12	1	13	10	7	13	5	-9	-5	-2	-2	3	-4	-4	-1	-1	-3	-1	-1	1	8	2	9	0	1	-2	0
	log2(1+Nuts)	25	-7	-3	12	13	10	11	21	20		8	9	3	4	2	7	4	2	5	4	-7	-2	-5	-1	-2	-2	-3	5	-1	0	-2	-2	-2	7	6	2	0	9	-2	1
	log2(Fruits)	25	42	-1	-39	-6	11	16	21	21	8		45	4	27	4	23	20	15	36	13	-17	-8	-7	-2	-2	-3	-3	4	2	3	-2	-6	-7	12	4	20	0	1	-3	0
	log2(Vegetables)	32	14	17	-20	8	24	28	20	28	9	45		6	20	4	19	13	20	12	7	-10	-6	-6	-2	-4	-3	-4	5	0	1	0	-3	-2	15	12	18	-2	7	-4	0
Blood nutrients	Retinol	-3	3	7	-9	1	0	4	2	2	3	4	6		10	9	5	6	11	9	37	-11	-12	4	3	40	23	9	1	21	8	3	9	-5	0	1	3	3	8	-1	2
	Mean carotenoids	0	16	6	-21	-16	3	8	2	12	4	27	20	10		56	79	79	71	75	29	-23	-31	-31	-20	-15	25	22	25	6	-9	-7	-26	-34	17	11	18	0	8	-13	-6
	Lycopene	2	-3	2	0	8	3	4	-1	1	2	4	4	9	56		28	29	30	25	11	4	-11	-12	-6	-3	24	22	12	1	-5	0	-9	-10	5	8	1	3	12	-3	-2
	log2(alpha-Carotene)	0	19	6	-22	-19	4	7	7	13	7	23	19	5	79	28		67	45	49	17	-25	-28	-29	-18	-17	11	11	19	6	-9	-5	-25	-31	19	7	17	-1	6	-11	-4
	log2(beta-Carotene)	-3	17	6	-20	-18	2	6	2	10	4	20	13	6	79	29	67		41	53	29	-32	-28	-28	-18	-20	14	15	18	7	-8	-9	-22	-31	13	2	16	0	3	-11	-6
	log2(Lutein+Zeaxanthin)	-2	8	7	-15	-15	4	10	-2	7	2	15	20	11	71	30	45	41		46	25	-10	-23	-24	-15	-7	28	21	25	5	-4	-5	-16	-28	13	13	14	0	13	-9	-4
	log2(beta-Cryptoxanthin)	2	19	-1	-20	-13	2	3	3	13	5	36	12	9	75	25	49	53	46		26	-22	-23	-20	-14	-8	18	15	17	2	-5	-5	-22	-24	13	6	15	3	6	-11	-6
	log2(alpha-Tocopherol)	-3	10	7	-12	-8	1	8	1	5	4	13	7	37	29	11	17	29	25	26		-54	-7	-2	-6	37	36	22	3	2	6	-4	2	-9	5	3	8	4	5	-6	-4
	log2(gamma-Tocopherol)	1	-14	-6	17	13	-3	-11	-6	-9	-7	-17	-10	-11	-23	4	-25	-32	-10	-22	-54		23	19	17	13	14	18	-20	1	8	9	16	22	-10	-7	-11	-6	-13	9	8
Measurements	log2(C-reactive protein)	6	-14	4	15	13	6	0	1	-5	-2	-8	-6	-12	-31	-11	-28	-28	-23	-23	-7	23		35	23	21	2	2	-19	-2	7	4	24	45	-4	-4	-15	1	-7	12	8
	log2(Insulin)	7	-13	3	16	17	7	-1	2	-2	-5	-7	-6	4	-31	-12	-29	-28	-24	-20	-2	19	35		42	37	-1	0	-39	4	16	10	40	51	-11	-7	-18	1	-14	11	7
	log2(Glucose)	3	-9	5	8	9	4	1	1	-2	-1	-2	-2	3	-20	-6	-18	-18	-15	-14	-6	17	23	42		21	1	1	-22	-4	16	7	26	29	-5	-6	-8	13	-6	6	6
	log2(Triglyceride)	2	2	1	2	7	1	-3	-1	3	-2	-2	-4	40	-15	-3	-17	-20	-7	-8	37	13	21	37	21		31	17	-49	5	14	7	32	21	-7	-6	-10	1	-9	7	5
	Total cholesterol	-6	1	-2	-3	-5	-2	2	-5	-4	-2	-3	-3	23	25	24	11	14	28	18	36	14	2	-1	1	31		93	8	10	7	2	5	-2	-3	-4	3	1	6	1	3
	LDL cholesterol	-6	2	-2	-3	-5	-3	1	-5	-4	-3	-3	-4	9	22	22	11	15	21	15	22	18	2	0	1	17	93		-9	10	4	2	5	1	-4	-5	1	1	4	1	3
	HDL cholesterol	-3	-4	-1	-2	-8	0	5	0	-1	5	4	5	1	25	12	19	18	25	17	3	-20	-19	-39	-22	-49	8	-9		-4	-4	-5	-31	-25	9	9	11	-1	20	-9	-4
	log2(Creatinine)	-1	5	-2	-3	-1	-2	-2	0	-1	-1	2	0	21	6	1	6	7	5	2	2	1	-2	4	-4	5	10	10	-4		4	3	0	-1	2	1	2	-1	1	2	1
	Systolic blood pressure	1	1	-2	1	2	-1	-2	0	-3	0	3	1	8	-9	-5	-9	-8	-4	-5	6	8	7	16	16	14	7	4	-4	4		54	15	13	-9	-6	-6	-1	-1	7	4
	Diastolic blood pressure	5	-4	-1	4	3	3	5	1	-1	-2	-2	0	3	-7	0	-5	-9	-5	-5	-4	9	4	10	7	7	2	2	-5	3	54		4	14	-2	2	-3	1	2	4	5
	log2(Waist / hip ratio)	6	-8	1	9	12	3	-1	2	-1	-2	-6	-3	9	-26	-9	-25	-22	-16	-22	2	16	24	40	26	32	5	5	-31	0	15	4		33	-10	-13	-9	0	-8	9	5
	BMI	11	-15	9	18	19	11	6	6	1	-2	-7	-2	-5	-34	-10	-31	-31	-28	-24	-9	22	45	51	29	21	-2	1	-25	-1	13	14	33		-8	-9	-18	1	-13	9	8
Socio-behavioral	Education	7	6	5	-11	-7	7	14	10	8	7	12	15	0	17	5	19	13	13	13	5	-10	-4	-11	-5	-7	-3	-4	9	2	-9	-2	-10	-8		37	11	1	17	-10	-2
	Income	4	-1	3	-5	1	9	14	3	2	6	4	12	1	11	8	7	2	13	6	3	-7	-4	-7	-6	-6	-4	-5	9	1	-6	2	-13	-9	37		5	2	23	-6	0
	log2(1+Exercise)	-1	13	9	-21	-12	1	9	6	9	2	20	18	3	18	1	17	16	14	15	8	-11	-15	-18	-8	-10	3	1	11	2	-6	-3	-9	-18	11	5		0	10	-7	-4
	Current smoker	0	-1	1	1	2	1	1	1	0	0	0	-2	3	0	3	-1	0	0	3	4	-6	1	1	13	1	1	1	-1	-1	-1	1	0	1	1	2	0		-3	-1	0
	log2(1+Alcohol)	7	-14	-2	-7	5	9	19	5	1	9	1	7	8	8	12	6	3	13	6	5	-13	-7	-14	-6	-9	6	4	20	1	-1	2	-8	-13	17	23	10	-3		-7	-2
AA	EEAA	-2	0	-3	2	2	-3	-7	-2	-2	-2	-3	-4	-1	-13	-3	-11	-11	-9	-11	-6	9	12	11	6	7	1	1	-9	2	7	4	9	9	-10	-6	-7	-1	-7		37
	IEAA	0	2	-2	0	3	-5	-2	0	0	1	0	0	2	-6	-2	-4	-6	-4	-6	-4	8	8	7	6	5	3	3	-4	1	4	5	5	8	-2	0	-4	0	-2	37	

Supplementary Figure 3. Related to Figure 1. Correlations between diet, biomarkers, and sociodemographic factors in the WHI. The percent correlations (biweight midcorrelation, -100 < %r < 100) between adjusted dietary and biomarker factors are colored according to their magnitude, with positive correlations in red, negative correlations in blue. Blood biomarkers were measured from fasting plasma collected at baseline. At 903 ≤ n ≤ 4200 samples, the Bonferroni corrected significance threshold corresponds approximately to a percent correlation of | %r | ≥ 10 (shown in bold). Food groups and nutrients are inclusive, including all types and all preparation methods, e.g. folic acid includes synthetic and natural, and dairy includes cheese and all types of milk. Variables have been adjusted for ethnicity and originating dataset (WHI BA23 or WHI AS315).

**A** EEAA n=922	Model 1 *Minimal* β	p	Model 2 *Food* β	p	Model 3 *Biomarkers* β	p	Model 4 *MetS* β	p	Model 5 *Full* β	p
log2(1 + Fish)			-1.45	0.05					-1.41	0.06
log2(1 + Poultry)			0.55	0.39					0.54	0.39
Mean carotenoids			-1.25	**5E-6**					-1.10	**1E-4**
Current drinker			-0.60	0.12					-0.52	0.18
Education	-0.33	**3E-4**	-0.24	**0.01**	-0.30	**1E-3**	-0.32	**5E-4**	-0.23	**0.02**
BMI	0.12	**9E-5**	0.07	**0.03**	0.03	0.36	0.09	**0.01**	0.02	0.55
Physically active	-0.17	0.71	0.14	0.76	-0.04	0.93	-0.17	0.71	0.21	0.64
Current smoker	0.65	0.23	0.26	0.63	0.50	0.36	0.61	0.26	0.25	0.65
African American	-3.22	**3E-14**	-3.13	**3E-13**	-3.43	**2E-14**	-3.19	**5E-14**	-3.29	**6E-13**
Hispanic	0.24	0.64	0.34	0.51	-0.05	0.92	0.26	0.62	0.09	0.86
log2(C-reactive protein)					0.32	**0.02**			0.24	0.08
log2(Insulin)					0.54	**0.03**			0.45	0.08
log2(Triglycerides)					-0.18	0.58			-0.01	0.97
log2(Glucose)					-0.75	0.22			-0.50	0.44
HDL Cholesterol					-0.03	0.06			-0.02	0.17
Systolic blood pressure					0.02	0.09			0.02	0.06
Diastolic blood pressure					-0.01	0.58			-0.01	0.55
log2(Waist-to-hip ratio)					-0.77	0.61			-1.22	0.43
Metabolic syndrome symptoms							0.27	0.07	-0.13	0.62

**B** IEAA n=922	Model 1 *Minimal* β	p	Model 2 *Food* β	p	Model 3 *Biomarkers* β	p	Model 4 *MetS* β	p	Model 5 *Full* β	p
log2(1 + Fish)			-0.34	0.57					-0.22	0.71
log2(1 + Poultry)			-0.59	0.25					-0.60	0.23
Mean carotenoids			-0.41	0.06					-0.40	0.07
Current drinker			0.17	0.58					0.16	0.59
Education	-0.15	**0.04**	-0.13	0.07	-0.13	0.08	-0.14	**0.05**	-0.12	0.12
BMI	0.08	**1E-3**	0.07	**0.01**	0.05	0.10	0.06	**0.02**	0.06	0.05
Physically active	-0.16	0.65	-0.06	0.87	-0.13	0.71	-0.16	0.65	-0.04	0.91
Current smoker	-0.16	0.72	-0.32	0.46	-0.13	0.77	-0.18	0.68	-0.23	0.61
African American	-0.80	**0.02**	-0.64	0.06	-0.69	**0.05**	-0.78	**0.02**	-0.52	0.15
Hispanic	-1.48	**4E-4**	-1.32	**2E-3**	-1.54	**3E-4**	-1.47	**4E-4**	-1.42	**1E-3**
log2(C-reactive protein)					0.08	0.46			0.04	0.70
log2(Insulin)					0.25	0.22			0.26	0.21
log2(Triglycerides)					0.54	**0.04**			0.76	**0.01**
log2(Glucose)					-0.39	0.42			-0.08	0.88
HDL Cholesterol					0.01	0.53			0.00	0.83
Systolic blood pressure					0.01	0.15			0.02	**0.05**
Diastolic blood pressure					-0.02	0.42			-0.01	0.46
log2(Waist-to-hip ratio)					0.19	0.88			0.38	0.76
Metabolic syndrome symptoms							0.15	0.21	-0.30	0.14

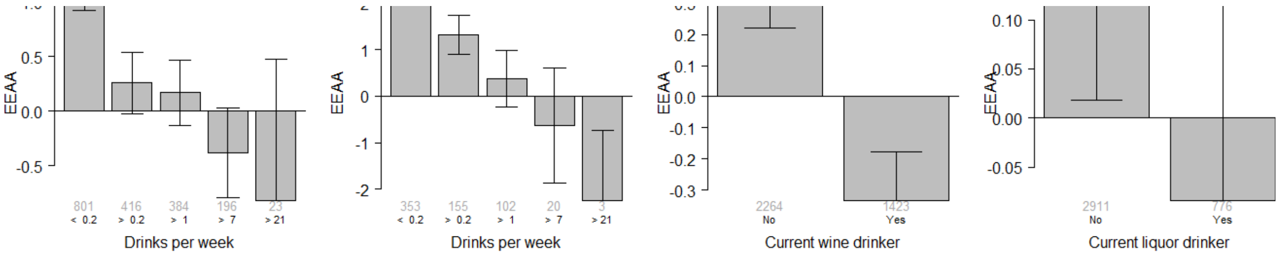

Supplementary Figure 4. EEAA among different strata of ethnic groups, levels and types of alcohol intake in the WHI. Panels **A**-**D** show bar plots visualizing the EEAA among stratified levels of alcohol intake (medium servings per week) for select ethnic groupings. Panels **E**-**H** show bar plots visualizing the EEAA among non- and current drinkers (at least one drink per month) of different types of alcoholic drinks: all types, beer, wine, and liquor. The sample sizes for each stratum are shown in grey beneath each bar. P-values for differences between strata are listed above each bar plot.

**A** EEAA	WHI Caucasian		African		Hispanic		Asian		Meta-analysis	
n	886		481		259		100		886	
	β	p	β	p	β	p	β	p	meta-t	meta-p
log2(1 + Fish)	-1.18	0.18	-0.99	0.28	-0.91	0.50	-0.67	0.73	-1.88	0.06
log2(1 + Poultry)	0.03	0.97	-0.03	0.98	0.47	0.65	1.82	0.35	0.41	0.68
Mean carotenoids	-0.98	**2E-3**	-0.93	**0.02**	-0.68	0.17	-1.25	0.16	-4.34	**1E-5**
Current drinker	-0.80	**0.04**	0.17	0.77	-0.95	0.18	-2.36	**0.04**	-2.31	**0.02**
Education	-0.05	0.62	-0.17	0.22	-0.47	**2E-3**	0.04	0.89	-2.20	**0.03**
BMI	0.03	0.43	0.14	**2E-3**	0.07	0.28	0.11	0.37	2.85	**4E-3**
Physically active	-0.54	0.28	0.47	0.44	1.06	0.19	-2.49	0.11	-0.24	0.81
Current smoker	1.15	0.06	0.75	0.35	-2.29	**0.02**	-0.82	0.76	0.87	0.38

**B** IEAA	WHI Caucasian		African		Hispanic		Asian		Meta-analysis	
n	886		481		259		100		886	
	β	p	β	p	β	p	β	p	meta-t	meta-p
log2(1 + Fish)	0.20	0.78	-0.90	0.24	-0.21	0.85	1.05	0.52	-0.35	[illegible]
log2(1 + Poultry)	-0.50	0.43	0.13	0.85	-0.84	0.30	-2.23	0.18	-1.20	[illegible]
Mean carotenoids	-0.62	**0.02**	-0.43	0.20	-0.14	0.71	0.24	0.75	-2.47	[illegible]
Current drinker	-0.27	0.40	0.42	0.38	-0.42	0.46	1.09	0.27	-0.16	[illegible]
Education	-0.02	0.80	-0.22	0.06	-0.06	0.63	-0.35	0.15	-1.72	[illegible]
BMI	0.04	0.22	0.05	0.19	0.04	0.41	0.36	**8E-4**	2.72	[illegible]
Physically active	-0.10	0.82	0.05	0.92	0.91	0.16	-2.26	0.09	0.02	[illegible]
Current smoker	-0.17	0.74	-0.70	0.30	-0.55	0.48	0.13	0.96	-1.05	[illegible]

Supplementary Figure 5. Related to Figure 2. Meta-analysis of multivariable linear models of EEAA and IEAA including carotenoid levels in the WHI. Analogous to Figure 2 except including mean carotenoid levels: EEAA (panel **A**) and IEAA (panel **B**) were regressed on potential confounding factors, fish and poultry intake, mean across standardized measures of carotenoids, and current drinker status for the ethnic strata with sufficient sample sizes (n>100). Individual columns correspond to coefficient estimates (β) colored blue or red for negative and positive values respectively, and p-values (p) colored in green according to magnitude of significance, with the exception of the last two columns which denote Stouffers's method meta-t and meta-p values. Models are adjusted for originating dataset (WHI BA23 or WHI AS315).

A

EEAA n=922	Model 1 Minimal β	p	Model 2 Food β	p	Model 3 Biomarkers β	p	Model 4 MetS β	p	Model 5 Full β	p
log2(1 + Fish)			-1.45	0.05					-1.41	0.06
log2(1 + Poultry)			0.55	0.39					0.54	0.39
Mean carotenoids			-1.25	**5E-6**					-1.10	**1E-4**
Current drinker			-0.60	0.12					-0.52	0.18
Education	-0.33	**3E-4**	-0.24	**0.01**	-0.30	**1E-3**	-0.32	**5E-4**	-0.23	**0.02**
BMI	0.12	**9E-5**	0.07	**0.03**	0.03	0.36	0.09	**0.01**	0.02	0.55
Physically active	-0.17	0.71	0.14	0.76	-0.04	0.93	-0.17	0.71	0.21	0.64
Current smoker	0.65	0.23	0.26	0.63	0.50	0.36	0.61	0.26	0.25	0.65
African American	-3.22	**3E-14**	-3.13	**3E-13**	-3.43	**2E-14**	-3.19	**5E-14**	-3.29	**6E-13**
Hispanic	0.24	0.64	0.34	0.51	-0.05	0.92	0.26	0.62	0.09	0.86
log2(C-reactive protein)					0.32	**0.02**			0.24	0.08
log2(Insulin)					0.54	**0.03**			0.45	0.08
log2(Triglycerides)					-0.18	0.58			-0.01	0.97
log2(Glucose)					-0.75	0.22			-0.50	0.44
HDL Cholesterol					-0.03	0.06			-0.02	0.17
Systolic blood pressure					0.02	0.09			0.02	0.06
Diastolic blood pressure					-0.01	0.58			-0.01	0.55
log2(Waist-to-hip ratio)					-0.77	0.61			-1.22	0.43
Metabolic syndrome symptoms							0.27	0.07	-0.13	0.62

B

IEAA n=922	Model 1 Minimal β	p	Model 2 Food β	p	Model 3 Biomarkers β	p	Model 4 MetS β	p	Model 5 Full β	p
log2(1 + Fish)			-0.34	0.57					-0.22	0.71
log2(1 + Poultry)			-0.59	0.25					-0.60	0.23
Mean carotenoids			-0.41	0.06					-0.40	0.07
Current drinker			0.17	0.58					0.16	0.59
Education	-0.15	**0.04**	-0.13	0.07	-0.13	0.08	-0.14	**0.05**	-0.12	0.12
BMI	0.08	**1E-3**	0.07	**0.01**	0.05	0.10	0.06	**0.02**	0.06	0.05
Physically active	-0.16	0.65	-0.06	0.87	-0.13	0.71	-0.16	0.65	-0.04	0.91
Current smoker	-0.16	0.72	-0.32	0.46	-0.13	0.77	-0.18	0.68	-0.23	0.61
African American	-0.80	**0.02**	-0.64	0.06	-0.69	**0.05**	-0.78	**0.02**	-0.52	0.15
Hispanic	-1.48	**4E-4**	-1.32	**2E-3**	-1.54	**3E-4**	-1.47	**4E-4**	-1.42	**1E-3**
log2(C-reactive protein)					0.08	0.46			0.04	0.70
log2(Insulin)					0.25	0.22			0.26	0.21
log2(Triglycerides)					0.54	**0.04**			0.76	**0.01**
log2(Glucose)					-0.39	0.42			-0.08	0.88
HDL Cholesterol					0.01	0.53			0.00	0.83
Systolic blood pressure					0.01	0.15			0.02	**0.05**
Diastolic blood pressure					-0.02	0.42			-0.01	0.46
log2(Waist-to-hip ratio)					0.19	0.88			0.38	0.76
Metabolic syndrome symptoms							0.15	0.21	-0.30	0.14

Supplementary Figure 6. Related to Figure 3. Multivariate linear models of EEAA and IEAA including carotenoid levels and with and without biomarkers in the WHI. Analogous to Figure 3 except including mean carotenoid levels: EEAA (panel **A**) and IEAA (panel **B**) were regressed on potential confounding factors, fish and poultry intake and current drinker status, and select biomarkers. Individual columns list the corresponding coefficient estimates (β) and p-values (p) for each fitting. Coefficients are colored according to sign (positive = red, negative = blue) and significance according to magnitude (green). Models 1 through 5 correspond to a minimal model, a model including dietary intake variables, a model including potential explanatory biomarkers, a model including number of metabolic syndrome symptoms and a complete model with all of the variables above, respectively. Models are adjusted for originating dataset (BA23 or AS315).

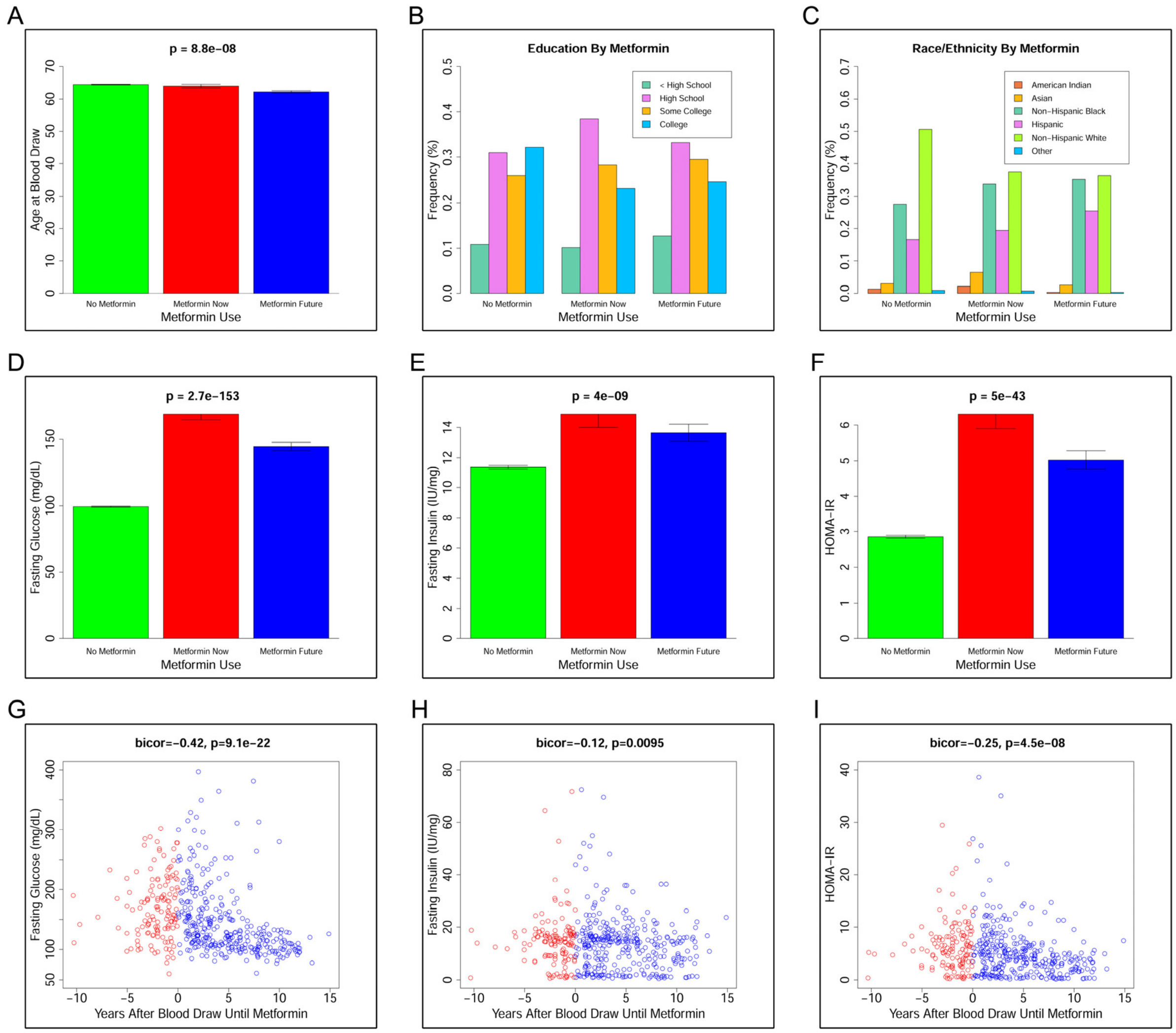

Supplementary Figure 7. Characteristics of non-, current, and future metformin users in the WHI. Panels **A-F** show barplots comparing the age at blood draw, educational attainment, ethnicity, fasting glucose, insulin, and insulin resistance of non-, current, and future metformin users (colored in green, red, and blue). Panels G-I show plotting fasting glucose, insulin, and insulin resistance as a function of years after blood draw until metformin with current and future users colored in red and blue respectively.

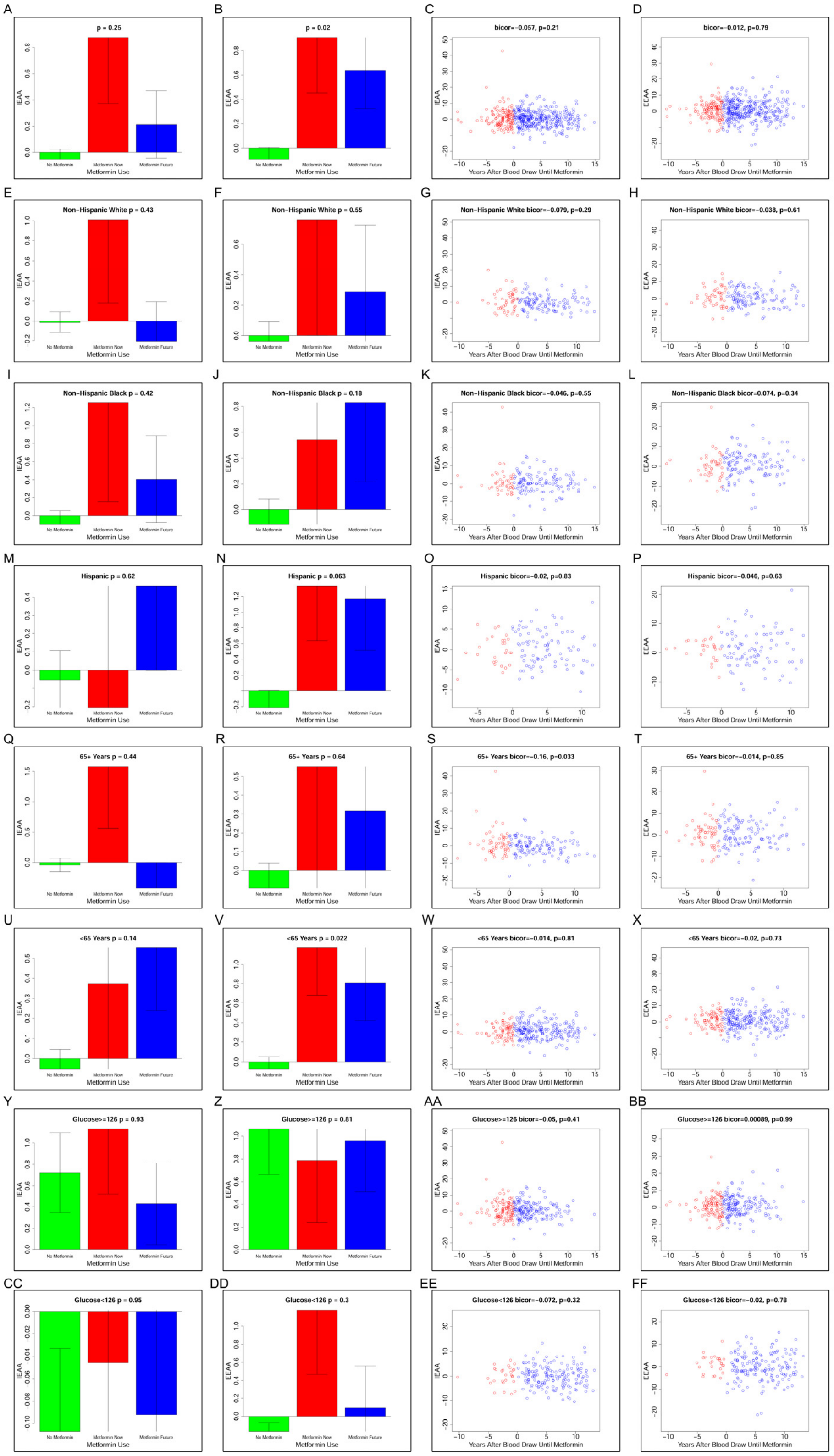

Supplementary Figure 8. IEAA and EEAA among non-, current, and future users of metformin in the WHI. Columns 1 and 2 show barplots with IEAA and EEAA on the y-axis and no metformin, current metformin, and later metformin usage strata (green, red, and blue respectively). Columns 3 and 4 show scatterplots with IEAA and EEAA plotted as a function of years after blood draw until metformin usage with current and future metformin users colored in red and blue respectively. Rows show the same plots for different sociodemographic strata: all available WHI participants, non-hispanic white, non-hispanic black, hispanic, > 65 years old, < 65 years old, hyperglycemic, and non-hyperglycemic groups. P-values (and correlation coefficients) are shown and the top of each plot.

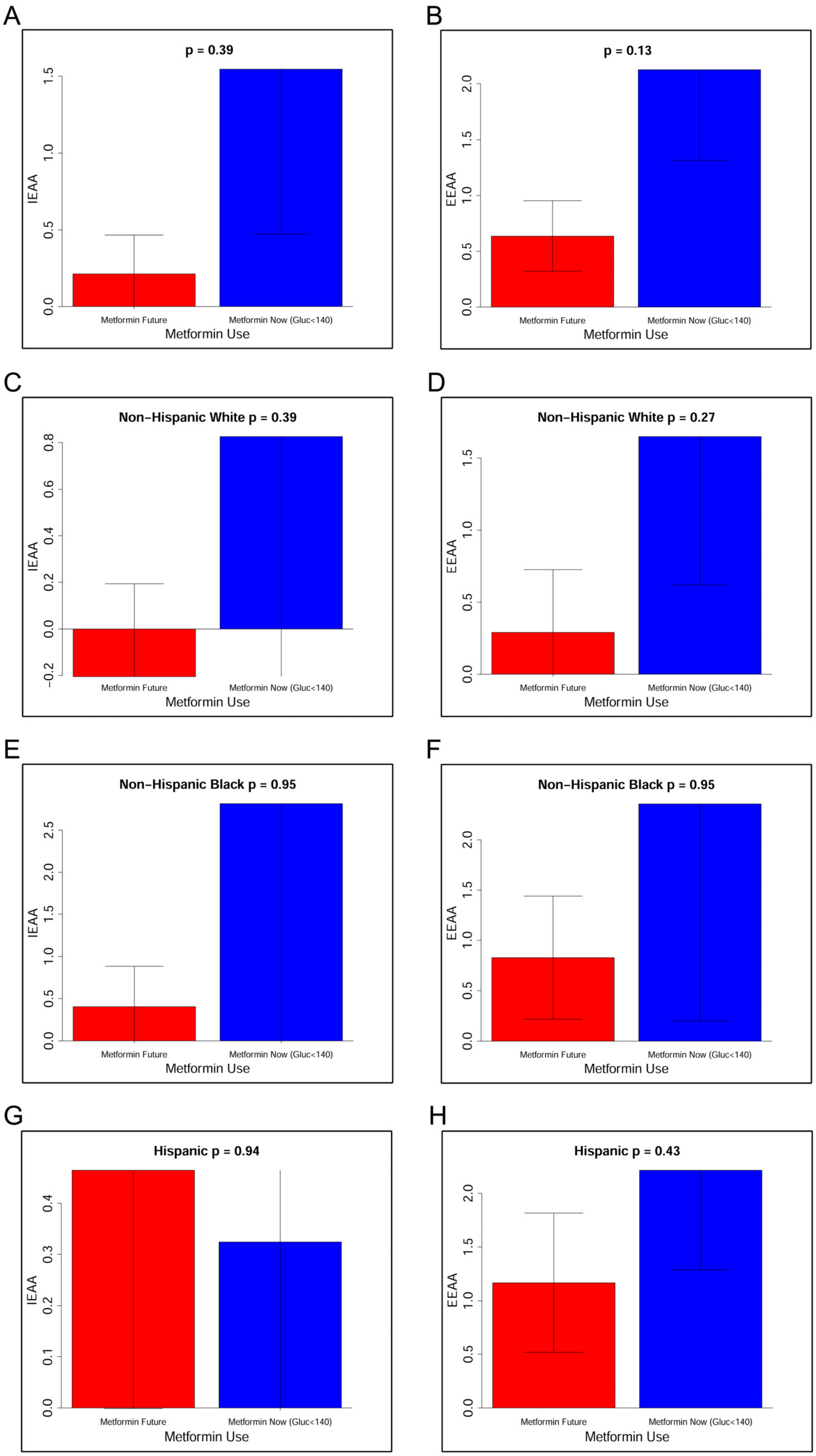

Supplementary Figure 9. IEAA and EEAA of future and non-hyperglycemic current metformin users in the WHI. Columns 1 and 2 show the IEAA and EEAA of future metformin users and current metformin users with fasting glucose levels of < 140mg/dL colored in red and blue respectively. Rows correspond to different ethnic strata: all ethnic groups, non-hispanic white, non-hispanic black, and hispanic. P-values for each comparison are shown at the top of each plot.

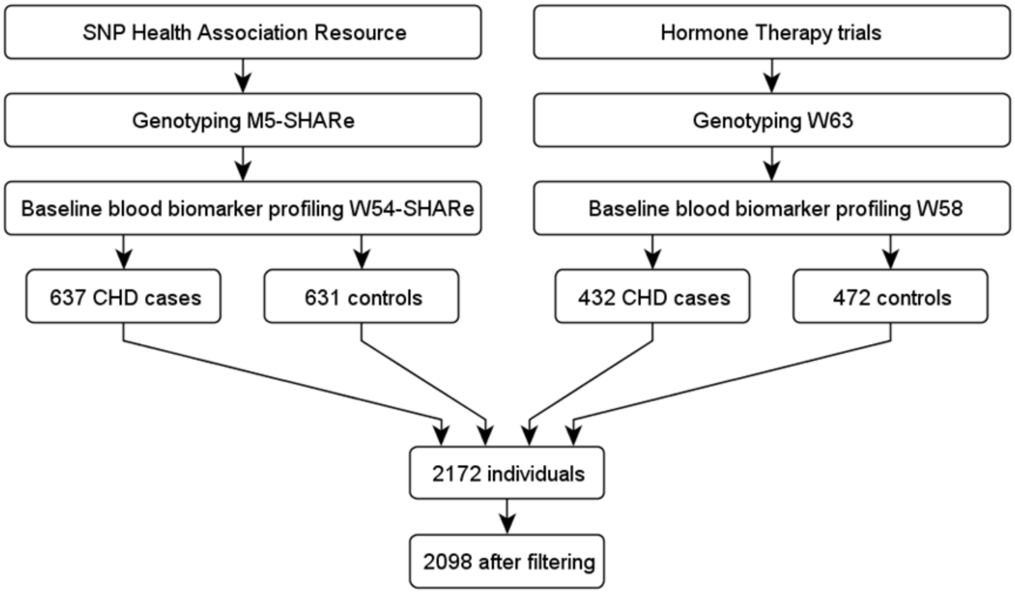

Supplementary Figure 10. Schematic denoting the selection of the WHI BA23 study sample. The study participants were originally selected for a case-control GWAS on coronary heart disease (CHD). Participants were selected from either the SNP Health Association Resource or the Hormone Therapy trials, and underwent genotyped and core blood biomarkers profiling. The data from these individuals were agglomerated and filtered for missing data.
